# Emotion processing in maltreated boys and girls: Evidence for latent vulnerability

**DOI:** 10.1007/s00787-022-02132-1

**Published:** 2023-02-04

**Authors:** Bianca Diaconu, Gregor Kohls, Jack C. Rogers, Ruth Pauli, Harriet Cornwell, Anka Bernhard, Anne Martinelli, Katharina Ackermann, Nikola Fann, Aranzazu Fernandez-Rivas, Miguel Angel Gonzalez-Torres, Maider Gonzalez de Artaza-Lavesa, Amaia Hervas, Christina Stadler, Kerstin Konrad, Christine M. Freitag, Graeme Fairchild, Pia Rotshtein, Stephane A. De Brito

**Affiliations:** 1 Centre for Human Brain Health, School of Psychology, University of Birmingham, Birmingham, B15 2TT UK; 2 Child Neuropsychology Section, Department of Child and Adolescent Psychiatry, Psychosomatics and Psychotherapy, University Hospital, RWTH Aachen, Aachen, Germany; 3 Department of Child and Adolescent Psychiatry, Faculty of Medicine, TU Dresden, Dresden, Germany; 4 Institute for Mental Health, School of Psychology, University of Birmingham, Birmingham, UK; 5 Department of Psychology, University of Bath, Bath, UK; 6 Department of Child and Adolescent Psychiatry, Psychosomatics and Psychotherapy, University Hospital Frankfurt, Goethe University, Frankfurt am Main, Germany; 7 School of Psychology, Fresenius University of Applied Sciences, Frankfurt am Main, Germany; 8 Psychiatric Service, Basurto University Hospital, Bilbao, Spain; 9 Faculty of Health Sciences, International University of La Rioja (UNIR), Logroño, Spain; 10 University Hospital Mutua Terrassa, Barcelona, Spain; 11 Global Institute of Neurodevelopment Integrated Care (IGAIN), Barcelona, Spain; 12 Department of Child and Adolescent Psychiatry, Psychiatric University Hospital, University of Basel, Basel, Switzerland; 13 JARA-Brain Institute II, Molecular Neuroscience and Neuroimaging, RWTH Aachen & Research Centre Juelich, Juelich, Germany; 14 Neuroimaging Research Unit, University of Haifa, Haifa, Israel

**Keywords:** Maltreatment, Psychopathology, Emotion processing, Sex differences, FemNAT-CD

## Abstract

Evidence of alterations in emotion processing in maltreated youth has been hypothesized to reflect latent vulnerability for psychopathology. However, previous studies have not systematically examined the influence of psychopathology on the results. Here, we examined emotion recognition and learning in youth who differed in terms of presence vs. absence of maltreatment and psychopathology and tested for potential sex effects. Maltreatment and psychopathology were assessed in 828 youth (514 females) aged 9–18 years using diagnostic interviews and self- and parent-report questionnaires. Emotion recognition was assessed via identification of morphed facial expressions of six universal emotions. For emotion learning, reward and punishment values were assigned to novel stimuli and participants had to learn to correctly respond/withhold response to stimuli to maximize points. A three-way interaction of maltreatment by psychopathology by emotion indicated that when psychopathology was low, maltreated youth were less accurate than non-maltreated youth for happy, fear and disgust. A three-way interaction of sex, maltreatment and emotion indicated that maltreated girls and boys were impaired for fear, but girls showed an impairment for happy, while boys for disgust. There were no effects of maltreatment, psychopathology, or sex on reward learning. However, a two-way interaction between sex and maltreatment showed that maltreated girls were worse at learning from punishment relative to non-maltreated girls, while maltreated boys were better than non-maltreated boys. The study provides the first clear evidence of latent-vulnerability in emotion recognition in maltreated youth and suggests that girls and boys might be characterized by distinct profiles of emotion recognition and learning following maltreatment.

**Supplementary Information:**

The online version contains supplementary material available at 10.1007/s00787-022-02132-1.

## Introduction

Childhood maltreatment refers to any act of omission (neglect) of care or commission (abuse) that results in actual or potential harm, regardless of intent [1]. Maltreatment is associated with negative cognitive, psychological and medical outcomes and is a reliable and important predictor of poor mental and physical health [2]. Maltreatment may alter certain developmental mechanisms such as those related to emotion processing, potentially leading to a cascade of negative consequences [3]. Indeed, these negative health outcomes have been hypothesized to be partly mediated by the numerous neurocognitive and neurobiological alterations associated with maltreatment that are thought to confer latent vulnerability [4–6]. While in the short term, these alterations may have adaptive value, especially in adverse environments, in the long term they can become maladaptive, leading to various psychiatric disorders across the lifespan [6]. The aim of the current study was to test for differences in emotion processing in youth with a history of maltreatment. We focused on facial expression recognition and reward/punishment emotion learning. Critically, we controlled for the presence of psychopathological symptoms, to ensure our findings are a true reflection of latent vulnerability in maltreated youth. Finally, we also explored sex differences in the way latent vulnerability is manifested.

Emotion recognition is the ability to recognize displays of emotions based on non-verbal information, such as facial expressions. Emotion recognition is an essential part of social communication. For example, accurate recognition of expressions serves as an important cue for trustworthiness or intent [7]. Research suggests that maltreatment is associated with altered emotion processing, but findings have been inconsistent [8]. A meta-analysis of studies assessing the ability to recognize facial expressions by children and adults with a history of maltreatment showed an overall impairment [9]. Though it is worth noting that out of 24 studies identified, seven reported impaired emotion recognition following maltreatment, three reported a superior emotion recognition, while 14 were excluded for lacking information about effect sizes. A qualitative integration of all 24 studies showed a more complex pattern. For example, one study reported that maltreated youth showed increased recognition for anger [10], fear and sadness [11], whereas others indicated reduced recognition for fear [12]. A more recent review [13] on facial emotion recognition in maltreated children reported that five of the nine studies indicated a reduced global emotion recognition in maltreated youth, with only one study [14] reporting a specific impairment for negative emotions. Finally, a recent meta-analysis [15] focusing on the recognition of sad, happy, fearful and angry expressions in individuals with adverse experiences (e.g., maltreatment, war, illness) before the age of 18 years showed that adverse events pre-adulthood were associated with impaired recognition for fear and happiness. Taken together, this literature suggests that maltreatment is associated with a reduced ability to recognize particular emotions, specifically fearful and happy expressions, but there is high heterogeneity across studies.

Emotion learning refers to the ability to adjust responses following feedback, which can be rewarding (positive) or ‘punishing’ (negative) and participants learn the reinforcement contingencies to maximize rewards and minimize punishment [16]. It is suggested that childhood maltreatment alters the learning environment through exposure to extreme parental affective reactions and inconsistency of reinforcers [17]. This can lead to unpredictable or extreme contingency learning and biased attention towards negative cues. A systematic review of the impact of early/childhood adverse effects on emotion learning in animals report that following maternal separation, rats and monkeys show weaker reward-based learning. Similarly, most of the research in humans to date has focused on the association between general adverse childhood experiences and stimulus-reinforcement learning, particularly reward-based learning [18]. For example, a study investigating eye movements in monetary incentive (reward-winning and punishing-losing) and non-incentive conditions showed that youth exposed to early-life stress (e.g., adoption, emotional neglect), exhibited slower responses than controls and failed to show reward incentive-related improvement on trials requiring inhibitory-saccade control [19]. Similarly, relative to typically developing youth, those with a history of adverse life events earned less points on reward-incentive trials; this was specifically observed for food insecurity but not neglect [20, 21]. Another study showed that adolescents exposed to early life stress are slower to learn positive and negative stimulus–response associations [22]. One longitudinal study showed that maltreatment and cumulative early adversity were associated with impaired punishment-based emotion learning and antisocial behavior [23]. However, this study did not examine responses to rewarding stimuli. Consistent with the above behavioral data, recent fMRI work has shown that early life stress exposure is associated with reduced rewards responsiveness in brain regions such as striatum, orbitofrontal, and medial frontal cortices, and increased response to punishment within the striatum, somatosensory and the lateral frontal cortices [24–26]. Taken together, the above studies suggest that maltreatment reduces reward-based learning, but possibly increases punishment-based learning. However, this is mostly based on the results of studies that have examined response to either reward or punishment separately, but not both components in the same task.

An important limitation for our understanding of how maltreatment impacts emotion processing is the high comorbidity of maltreatment with psychopathology. Indeed, maltreatment is associated with internalizing and externalizing psychopathology, which have themselves been linked to emotion recognition [11, 27] and learning deficits [27, 28]. Only a few behavioural studies have examined both maltreatment and psychopathology and how they relate to emotion recognition [15] and learning [18, 19] in the same individual. The meta-analysis [15] examining the impact of early adverse effects on expression recognition, outlines that the results from studies who report psychiatric diagnosis of the maltreated participants did not differ from those who did not. Similarly, for emotion learning, the presence of psychopathology symptoms did not affect the responses of youth with adverse history to incentive trials [19–21]. However, in most of the above studies maltreatment and psychopathology were often completely overlapping, making it impossible to disentangle the source of the deficits. One way to clarify the respective effects of maltreatment from psychopathology is to separate youth who have been exposed to maltreatment into those with and without psychopathology symptoms. This would enable to answer the question, of whether in the absence of overt psychopathological symptoms childhood maltreatment scar individuals, making them more vulnerable. According to the theory of latent vulnerability [4–6], maltreatment is associated with alterations in various neurobiological systems, which are thought to support short-term functional adaptation in the context of maladaptive environments. However, in the long term these alterations are associated with poor optimization to more adaptive environments, conferring risk to poor mental health. This means that it is possible for an individual to show resilience (no psychopathological symptoms) at one point in time, but still exhibit system alterations (i.e., latent vulnerabilities) that could, at a later point in time become detrimental for social functioning. As such, clarifying whether these alterations exist in maltreated youth who are resilient would enhance our understanding of the specific candidate neurocognitive systems that may increase vulnerability following maltreatment [6].

Beyond psychopathology, maltreatment can also have differential sex effects on emotion processing. There is a large body of evidence suggesting the two sexes differ in the way they respond to stress [29, 30]. Furthermore, studies from healthy individuals show that females are better at emotion recognition than males [31], and males outperform females in emotion learning [32]. However, the potential influence of sex on the association between maltreatment and emotion recognition/learning is understudied because of small samples providing insufficient statistical power. This is a major limitation given that sex has been shown to impact both the nature and severity of psychiatric outcome following maltreatment. Specifically, maltreatment-related psychiatric disorders are associated with a greater prevalence of internalizing psychopathology in females, and greater prevalence of externalizing psychopathology in males [33]. While some evidence suggest that maltreatment has a more detrimental effects on males [34, 35], other suggest a stronger effect on females [36].

The current study aims to revisit the impact of maltreatment on emotion processing by explicitly considering the influence of psychopathology and sex. We divided participants into four groups based on presence/absence of maltreatment history and psychopathology symptoms and used the emotion hexagon task [37] for assessing facial emotion recognition, and the passive avoidance learning task [38] as an index of emotion learning.

Given the available literature, we formulated the following hypotheses:

i. For emotion recognition, we expected to replicate the findings from the latest meta-analysis and find maltreatment to be associated with reduced recognition for both negative (fear) and positive (happiness) emotions [15].
ii. For emotion learning, we expected to observe disrupted reward-based learning in maltreated youth (i.e., more omission errors) and increased punishment learning (i.e., less commission errors).
iii. For both tasks, based on the latent vulnerability hypothesis, we expected to observe differences between the resilient (i.e., maltreated, low psychopathology) and the control group.
iv. Given evidence that psychopathological profiles associated with maltreatment differ between the sexes, we hypothesized that this would manifest as different profiles of emotion processing. However, we did not formulate a specific hypothesis regarding the direction and nature of the differences.

## Methods

### Participants and measures

The FemNAT-CD study [39] (*N* = 1827) included 11 sites across Europe. Participants were recruited via community outreach, mental health clinics and youth offending services, with an effort to optimize recruitment of females. Participants were recruited if they were within the age of 9–18 years. Participants with an IQ < 70 or with a diagnosis of autism spectrum disorder, schizophrenia, neurological conditions, and genetic syndromes were excluded. Typically developing participants were excluded if they had any psychiatric diagnosis. For the current study, 828 youth (514 females) aged 9–18 years (*M* = 13.8; SD = 2.5) completed the emotion hexagon task [37]. (See Supplement 1 for the distribution by group, sex and site and Supplement 2 for inclusion criteria). Of these, 717 youth (446 females) also completed the emotion learning task [38]. (See Supplement 3). Written informed consent/assent was obtained from all participants and their parents according to site-specific ethical requirements (see Supplements 4–6).

Consistent with previous maltreatment research [40], youth were divided into maltreated and non-maltreated groups using the Children’s Bad Experiences Questionnaire (CBE). The CBE is a 5-item semi-structured interview in which the informant (parent, caregiver, or guardian) is asked to provide information about the child’s experiences of physical, psychological, and sexual harm. The interviewers did not ask questions about the perpetrator of abuse, with a focus on whether these forms of maltreatment had been experienced by the child or not. The informant had the option to answer these questions with “Never”, “Yes”, “Frequent” or “I don’t know/Refuse to Answer”, followed by a detailed description of the event whenever applicable. Finally, based on all the information collected, a decision was made as to whether no/probable or definite maltreatment had been reported. In line with previous research using this instrument, participants were classified as ‘maltreated’ if probable or definite maltreatment was reported (See Supplement 7).

Psychopathology was assessed via the Child Behaviour Checklist (CBCL) [41] and the Kiddie Schedule for Affective Disorders and Schizophrenia for School-Age Children: Present and Lifetime Version (K-SADS-PL, see Supplement 8) [42]. The CBCL is a checklist completed by parents/caregivers to examine eight dimensions: anxiety/depression, withdrawal, somatic complaints, thought problems, attention problems, rule-breaking behavior, and aggressive behavior. Overall scores were used for analysis, with T scores > 65 as the clinical cut-off point [43]. The K-SADS-PL is a semi-structured diagnostic interview used to assess current and past psychopathology in children and adolescents (See Supplement 9 for inter-rater reliability information). Based on the above we classified participants to four groups: (1) control group: low psychopathology no maltreatment; (2) maltreatment with low psychopathological symptoms (resilient group); (3) high psychopathology with no history of maltreatment: and (4) high psychopathology with a history of maltreatment (See Table 1, for demographic details and characteristics of each group).

**Table 1:** Participants’ Demographic Characteristics and Psychopathology Subscales Scores

	Control	Resilient	High psychopathology	Psychopathology + Maltreatment	Group effects
Sample	*N* = 516	*N* = 30	*N* = 172	*N* = 110	*F*/*x*^2^
Age, M (SD)	13.7 (2.6)	13.9 (2.7)	13.9 (2.4)	13.8 (2.3)	0.4
Females (in %)	62.8	70.2	57.8	59.7	2.7
Estimated full-scale IQ, M (SD)	105.4 (11.7)^a^	104.3 (13.9)^a^	97.9 (11.6)^b^	98.4 (13.7)^b^	26.6***
Estimated verbal IQ, M (SD)	105.3 (14.9)^a^	103.2 (18.9)^a^	96.6 (13.6)^b^	95 (17.4)^b^	26.6***
Estimated performance IQ, M (SD)	104.8 (13.7)^a^	104.8 (13.5)^a^	98.7 (14.4)^b^	101.3 (14.8)^a^	10.9***
PDS (1 = pre/early puberty; 2 = mid/late/post puberty) (in %)	2–81.3	2–75.6	2–80.2	2–82.2	0.6
SES M (SD)	0.3 (0.9)^a^	0.2 (1.1)^a^	− 0.2 (1.1)^b^	− 0.4 (1.1)^b^	29.5***
CBCL total *t* Scores M (SD)	48.1 (8.5)^a^	52.4 (7.8)^a^	73.9 (6.5)^b^	75.1 (6.1)^b^	838.8***
CBCL internalizing scale	47.7 (8.3)^a^	53.8 (8.7)^a^	70.3 (11.9)^b^	74.4 (7.9)^c^	480.2***
CBCL externalizing scale	49.8 (8.5)^a^	53.5 (8.4)^a^	68.2 (8.6)^b^	70.3 (8.4)^b^	367.8***
CBCL anxiety/depression	53.3 (5.1)^a^	55.4 (6.4)^a^	65.5 (10.7)^b^	69.6 (9.6)^b^	242.3***
CBCL withdrawal	53.6 (5.3)^a^	55.1 (5.5)^a^	65.1 (10.2)^b^	67.7 (9.0)^b^	206.3***
CBCL somatic complaints	54.6 (5.8)^a^	57.1 (6.9)^a^	64.3 (10.1)^b^	64.9 (11.1)^b^	110.2***
CBCL social problems	52.7 (4.7)^a^	54.9 (6.6)^a^	65.8 (10.2)^b^	68.8 (9.8)^b^	273.1***
CBCL thought problems	52.3 (4.6)^a^	53.4 (5.8)^a^	66.4 (10.4)^b^	66.2 (10.3)^b^	263.9***
CBCL attention problems	52.9 (5.1)^a^	54.3 (4.9)^a^	68.5 (10.2)^b^	71.9 (10.1)^b^	375.8***
CBCL rule-breaking behavior	52.5 (4.5)^a^	54.8 (5.4)^a^	70.2 (11)^b^	74.4 (11.9)^b^	496.1***
CBCL aggressive behavior	52.6 (4.5)^a^	56.2 (6.6)^a^	72.2 (11.8)^b^	75.5 (10.6)^b^	496.1***

Control = no maltreatment, low psychopathology; Resilient = probable/definite maltreatment, low psychopathology; High psychopathology = no maltreatment, high psychopathology; Psychopathology + Maltreatment = high psychopathology, probable/definite maltreatment; *SES* = socioeconomic status (SES was computed based on parental income, education level and occupation); *PDS* = pubertal developmental status; *CBCL* = child behavior checklist; The CBCL scores for the Internalizing scale were computed using the Anxiety/Depression, Withdrawal and Somatic Complaints subscales, whereas the scores for the Externalizing scale were computed using the Rule-breaking and Aggressive Behavior subscales; Post hoc tests are reported based on observed means, where groups marked with different letters differ significantly from each other at *p <.05, **p<.01 and ***p<.001

Lastly, similar to our previous work [44], puberty status was assessed using the Pubertal Development Scale (PDS, Supplement 10) [45], and IQ was estimated using the Wechsler Intelligence Scales (WASI, WAIS, WISC) [46]. In English-speaking sites, IQ was estimated with the vocabulary and matrix reasoning subscales of the WASI-I. Other sites used the vocabulary, block design and matrix reasoning tests of the WISC (for participants aged ≤ 16) or WAIS (for participants aged 17–18 years).

### Experimental paradigms and dependent variables

**Fig. 1:**
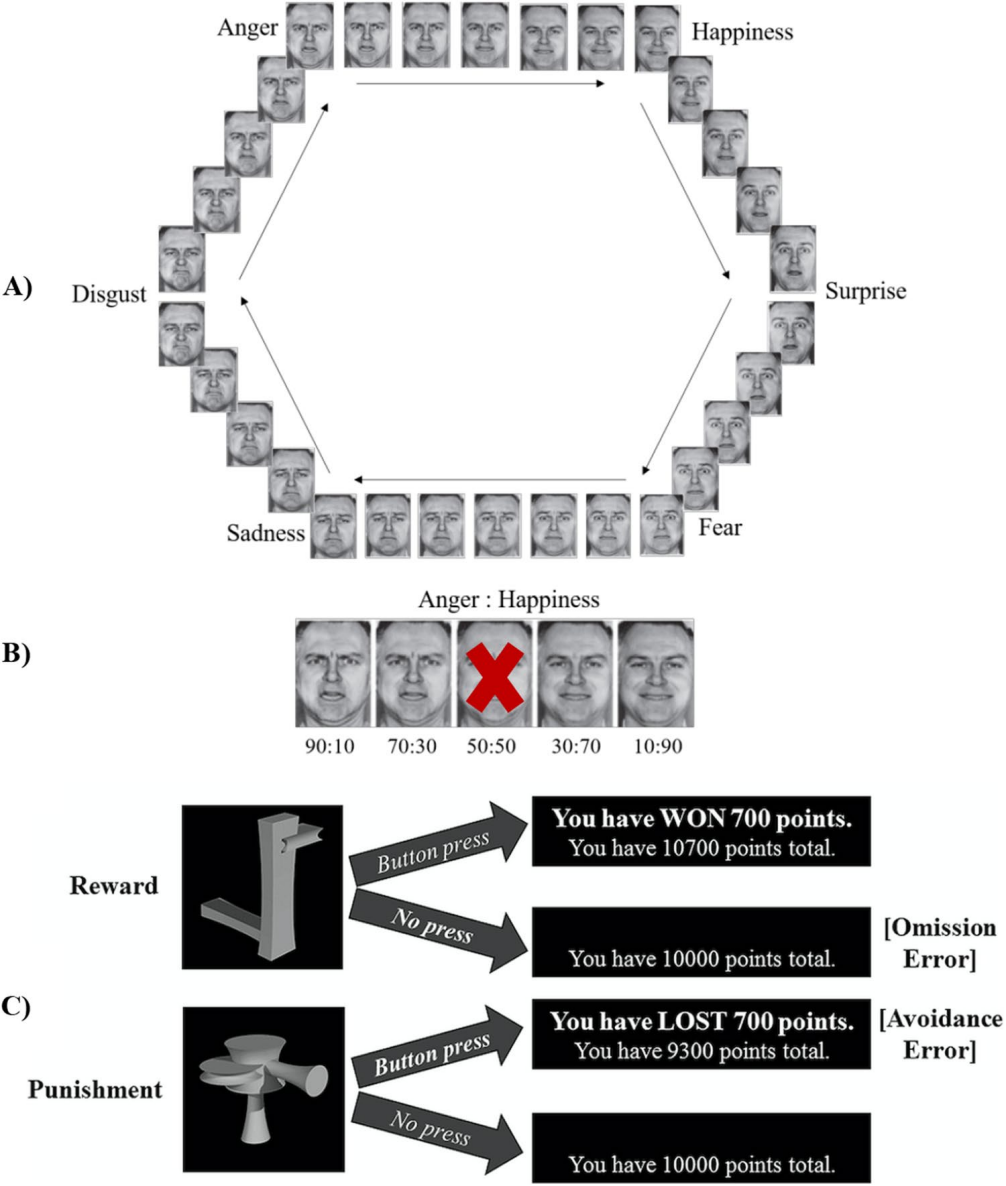
Schematic representations of the emotion hexagon and the passive avoidance learning tasks. **A)** The complete set of blended expressions arranged in a hexagon. The six basic emotions (anger, happiness, surprise, fear, sadness, and disgust) lie on the vertices adjacent to their most easily confused emotion. The faces on the edges of the hexagon are the blended expressions used as task stimuli. **B)** An example of the blended expressions for the anger-to-happiness continuum, with anger: happiness ratios labelled. Each continuum was presented with percentages of expressed emotions varying from 10 to 90% (e.g., 10% surprise–90% happiness, 30% surprise–70% happiness). Stimuli were presented using EPrime on a computer monitor in random order for three seconds and participants were asked to select the label that best described the emotion presented. The ‘dominant’ emotion (i.e., 90% or 70% present) was considered the correct response. Response time was not constrained, and no feedback was provided. The task included six blocks, each containing two 90% and two 70% expression morphs for each emotion, resulting in four correct instances of each expression per block. The first block was for practice, leaving only five blocks for analysis. Participants took approximately 20 min to complete this task. The 50–50% morphs were fillers and not scored. **C)** A schematic representation of the Passive Avoidance Learning Task using novel ‘ziggerin’ stimuli. Four stimuli were associated with reward and four with punishment fixed values (1, 700, 1400, 2000 points). Each stimulus was shown once within a block of 8 trials, with 10 blocks overall (including one practice block, excluded from analysis). Stimuli were displayed on a computer monitor for a maximum of 3 s, followed by performance feedback (i.e., points won or lost or no change, as well as the running total). Participants started the task with 10,000 points. The average completion time for this task was 5 min

Emotion recognition accuracy (in percent) of facial expressions was assessed using the Emotion Hexagon task, [37] including happy, sad, angry, fearful, disgusted, and surprised expressions. The dependent variable for this task was accuracy of emotion recognition (in %), for the dominantly presented emotion (e.g., 70% or 90% anger). For emotion learning, we used a modified Passive Avoidance Learning task [38] where participants had to learn by trial-and-error to respond to stimuli eliciting rewards (winning points) and to avoid responding to stimuli eliciting punishments (losing points). Incorrect responses to punishment stimuli were counted as commission errors and missed responses to reward stimuli were counted as omission errors. More details on the test battery can be found in Fig. 1. (See Supplement 11 for details on task randomization).

### Statistical analyses

For the Emotion Hexagon task, we used a 2 (Maltreatment: no vs. probable/definite) by 2 (Psychopathology: low vs. high) by 2 (Sex: male vs. female) by 6 (Emotions: anger, happiness, surprise, fear, sadness, and disgust) design. For the Passive Avoidance task, the design was a 2 (Maltreatment: no vs. probable/definite) by 2 (Psychopathology: low vs. high) by 2 (Sex: male vs. female) by 4 (Magnitude: 1, 700, 1400 or 2000 points) for both omission and commission errors. Repeated measures analyses of covariance (rmANCOVA) were used to analyze the accuracy data, with mean centered IQ, SES (See Supplement 12) and pubertal status (pre/early puberty vs. mid/late/post puberty) as covariates of no interest. To account for differences between sites of data collection, we re-run the analyses by including the sites with the largest number of participants (i.e., Germany and United Kingdom, *N* = 576; Supplement 13). Significant main effects of interactions were followed by pair-wise post hoc comparisons using Bonferroni correction. Effect sizes are reported as partial eta squared (*η*_*p*_^2^), interpreted as follows: small ≥ 0.01; medium ≥ 0.06; large ≥ 0.14 [47].

## Results

### Emotion recognition

For simplification, we only report significant main effects and interactions involving maltreatment, psychopathology, and sex, as per our aims. (See Supplements 14–15 for pubertal status and age results). For accuracy, the rmANCOVA revealed significant main effects of psychopathology, maltreatment, and sex (Table 2). Both maltreatment and psychopathology were associated with significantly decreased accuracy and females were significantly more accurate than males.

**Table 2:** Main and interactive effects of maltreatment, psychopathology and sex on emotion recognition and learning

		**Emotion recognition**			
*Main effects*	*F*	*df*	*p*	*η*_*p*_^2^	*Post hoc comparisons*
Maltreatment	3.94	1,808	0.047	0.005	–
Psychopathology	5.45	1,808	0.02	0.007	–
Sex	20.93	1,808	< 0.001	0.025	–
*Two-way interactions*					
Emotion by maltreatment	2.41	5,4040	0.034	0.003	**Fear**: maltreated < non-maltreated
					Anger, happiness, surprise, sadness, disgust: n.s
*Three-way interactions*					
Emotion by maltreatment by psychopathology	2.25	5,4040	0.047	0.003	Low psychopathology:
					**Happiness, fear, disgust**:
					Maltreated < non-maltreated
					Anger, surprise, sadness: n.s
					High psychopathology: n.s
Emotion by sex by maltreatment	2.98	5,4040	0.011	0.004	Females:
					**Fear, happiness**:
					Maltreated females < non-maltreated females
					Males:
					**Fear, disgust**
					Maltreated males < non-maltreated males

		**Emotion learning**			
*Main effects*	*F*	*df*	*p*	*η*_*p*_^2^	*Post hoc comparisons*
Psychopathology	8.98	1,707	0.003	0.013	–
Sex	4.83	1,707	0.028	0.007	–
*Two-way interactions*					
Sex by Maltreatment	4.68	1,707	0.031	0.007	Maltreated females > non-maltreated females
					Maltreated males < non-maltreated males
Sex by psychopathology	5.23	1,707	0.022	0.007	High psychopathology males > low psychopathology males
					Females: n.s

For Emotion Learning, the rmANCOVA also yielded significant two-way interactions between sex and block and three-way interactions between sex, block and maltreatment, and sex, block and psychopathology, respectively. For simplicity, we have not reported these results here, but they can be found in Supplement 17. However, the overall pattern of results showed that maltreated females made more avoidance errors than maltreated males during blocks 3, 6, 7, and 8, but by the 10th block, no significant sex differences were observed anymore. Interestingly, pairwise comparisons in the sex by block by psychopathology interaction showed that low psychopathology females made more avoidance errors during blocks 3, 6, 7, 8 than low psychopathology males, but no significant sex differences were found in any blocks when psychopathology was high. These analyses echo the rest of our reported results whereby maltreated females showed the most impaired performance on the emotion learning task. Covariates evaluated in the model were mean-centered SES = 0.125, IQ = 1.245, and pubertal status (1 = pre/early puberty; 2 = mid/late/post puberty). The adjustment for multiple comparisons were obtained using the Bonferroni correction

**Fig. 2:**
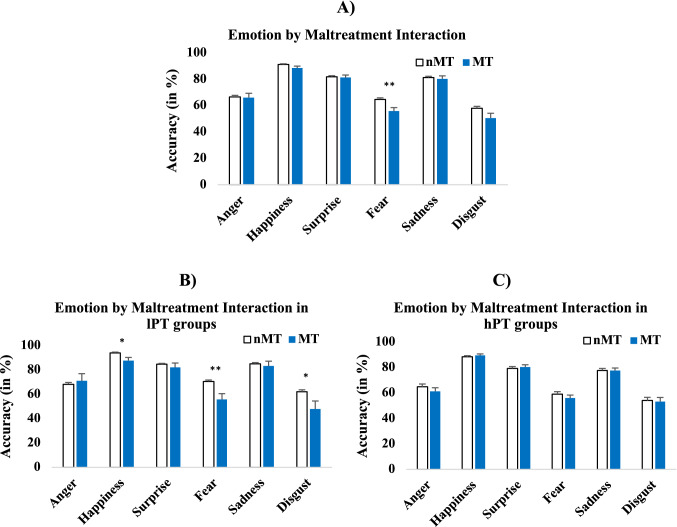
**A)** Interaction between emotion and maltreatment for recognition accuracy; maltreated (MT) youth are significantly less accurate than non-maltreated (nMT) youth when recognizing fear (collapsing across the high and low psychopathology groups). **B)** Percentage accuracy across emotions between the nMT and MT groups when psychopathology was low (lPT); MT youth were significantly less accurate than nMT for happiness, fear and disgust. **C)** Percentage accuracy across emotions between the nMT and MT groups when psychopathology was high (hPT); Here, no significant differences were found between the nMT and the MT groups regardless of emotion. Groups differ significantly at *p* < 0.05 level (*), *p* < 0.01 (**), and *p* < 0.001 level. All error bars show ± 1 standard error of the mean

There were also significant two-way interactions between emotion and maltreatment, and a three-way interaction between emotion, maltreatment, and psychopathology. For the two-way interaction, post-hoc analyses indicated that maltreated youth were significantly less accurate than non-maltreated youth when recognizing fear, but no significant differences were found for the other emotions (Fig. 2a). However, for the three-way interaction, post hoc analyses showed that when psychopathology levels were low, maltreated youth were significantly less accurate than non-maltreated youth when recognizing happiness, fear, and disgust. When psychopathology levels were high, no significant differences were found between maltreated and non-maltreated youth for any emotion (Fig. 2b, c).

**Fig. 3:**
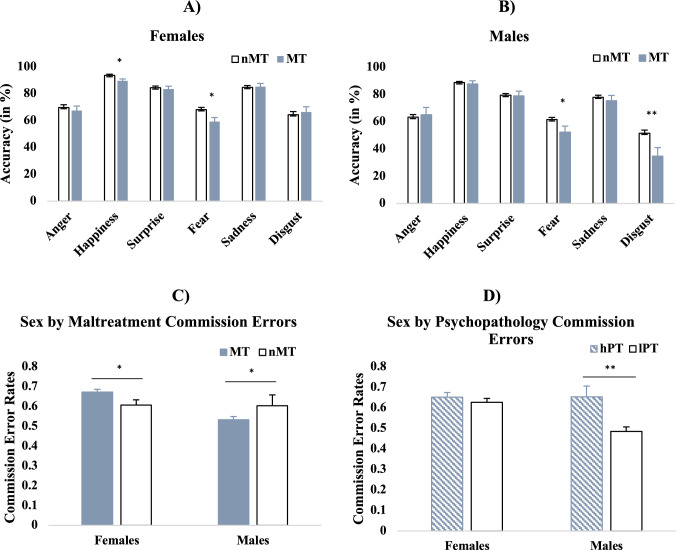
**A)** Interaction between sex, maltreatment and emotion on recognition accuracy; This panel shows the effects of MT in females; here, MT females are significantly less accurate than nMT females for happiness and fear. **B)** This panel shows MT effects in males; here, MT males are significantly less accurate than nMT males for fear and disgust. **C)** Commission error rates by sex and maltreatment. Here, maltreated females made significantly more commission errors than non-maltreated females, whereas maltreated males made less errors than nonmaltreated males. **D)** Commission errors rates by sex and psychopathology. Females with low psychopathology did not significantly differ in their avoidance errors compared to females with high psychopathology, but high psychopathology males made more errors than low psychopathology males. Groups differ significantly at *p* < 0.001 (***), *p* < 0.01 (**), and *p* < 0 .05 level (*). All error bars show ± 1 standard error of the mean

Lastly, we also found a three-way interaction between sex, emotion and maltreatment. Post-hoc analyses indicated that maltreated females were less accurate for happiness and fear compared to non-maltreated females. Maltreated males were less accurate for fear, and disgust compared to non-maltreated males. (Fig. 3a, b) (See Supplement 16 for additional analyses on females only).

### Emotion learning

For commission errors, the rmANCOVA revealed significant main effects of psychopathology, and sex (Table 2). Specifically, high-psychopathology youth made significantly more commission errors than low-psychopathology youth and females made significantly more errors than males. Significant two-way interactions were found between sex and maltreatment, and sex and psychopathology. Post-hoc analyses indicated that maltreated females made significantly more errors than non-maltreated females, whereas the opposite pattern was observed for males, where the non-maltreated males made more commission errors than the maltreated group. (Fig. 3c, d). Lastly, high and low psychopathology females did not significantly differ, but high psychopathology males made more errors than low psychopathology males (Table 2). For the results by block, please see Supplement 17.

For omission errors, the rmANCOVA did not yield any significant main effects for either maltreatment or psychopathology. Similarly, no interactions were found between any of the other variables.

## Discussion

Our study investigated emotion recognition and learning in maltreated and non-maltreated youth with and without psychopathology to address two aims. First, we wanted to clarify whether maltreated youth exhibit abnormities in emotion recognition and learning and to what extent the presence or absence of psychopathology would influence their neurocognitive profile. We hypothesized that maltreated youth would show reduced emotion recognition for fear and happiness. Our findings partially supported this, showing that maltreatment was specifically associated with a lower recognition of fear. Crucially, however, maltreatment, psychopathology and emotion interacted such that youth exposed to maltreatment and low levels of psychopathology showed deficits in happiness, fear and disgust recognition versus non-maltreated youth with low psychopathology. No differences between maltreated and non-maltreated youth were found when psychopathology was high. These findings support our third hypothesis suggesting a latent vulnerability effect for emotion recognition. For emotion learning, we hypothesized that maltreated youth would show reduced reward learning and increased punishment learning. This hypothesis was, however, not supported as indicated by the absence of significant main effects of maltreatment for reward-based learning and only a main effect of psychopathology for punishment-based learning; youth with high psychopathology made more commission errors than youth with low psychopathology. Second, we investigated whether sex interacted with maltreatment and/or psychopathology to predict emotion recognition and learning and hypothesized that maltreated males and females would exhibit different profiles of emotion processing. Consistent with that hypothesis, for emotion recognition we showed that maltreatment in females was associated with poorer recognition of happiness and fear, while in males, it was associated with a lower recognition of fear and disgust. For emotion learning, maltreatment was associated with more commission errors in females, but less commission errors in males.

The lower accuracy for both fear and happiness is consistent with the results of a recent meta-analysis of 29 studies (20 on youth aged 4.4–17.5) showing that maltreatment, particularly when experienced before the age of 3, was associated with reduced accuracy for those emotions, but not for sad or anger; unfortunately no data were available for disgust [15]. However, our study is the first to show that maltreated youth who appear resilient on the surface might in fact exhibit a latent vulnerability in the form of lower emotion recognition for both negative (fear and disgust) and positive (happiness) basic emotions. Interestingly, and in contrast to our study, the above meta-analyses did not find a moderating effect of psychopathology [15]. In terms of potential mechanisms, happy faces have been shown to facilitate social bonding and affiliation and are thus considered a form of social reward stimulus because they signal positive emotions, attachment availability, care, support, which all contribute to the development of trust [48, 49]. Indeed, there is now good evidence indicating that trust is a prerequisite for successful social relationships and facial expressions are one of the main sources of information when forming an impression about someone’s trustworthiness [50]. Recent research suggests that relative to their peers, maltreated children are less likely to rate unfamiliar faces as trustworthy, which is thought to contribute to a reduced social network [7, 51]. Thus, consistent with the latent vulnerability hypothesis, we speculate that the lower accuracy for happy faces in the resilient group indexes as a latent vulnerability, which long-term might reduce social affiliations via reduced trust, potentially increasing the risk for future psychopathology. The resilient group also exhibited lower accuracy for fear and disgust faces, which, like happy faces, are reinforcers, but associated with potential threat and signaling that someone or something should be avoided [52]. The reduced accuracy for those two negative facial expressions could thus reflect an avoidance bias. Indeed, previous studies investigating attention processes to threat in maltreated samples have identified an avoidance in processing fear, which may be caused by prolonged exposure to threatening or chaotic environments [53, 54]. This interpretation is supported by our additional analyses (see Supplement 18) on the confusability of emotion responses, which indicated that compared to controls, maltreated youth with low psychopathology (i.e., resilient) showed more bias towards disgust when fear was depicted. However, further research using eye-tracking would be needed to clarify whether our results do indeed reflect avoidance of these emotions.

In terms of emotion learning, contrary to our second and third hypotheses, we did not find a main effect of maltreatment on reward/punishment learning nor did we find that maltreatment and psychopathology interacted (i.e., no latent vulnerability). However, it is important to note that most behavioural studies reporting impaired reward learning investigated youth with experiences of early life stress (i.e., cumulative adverse experiences, not just sole maltreatment) [17, 22]. Thus, our study adds to the literature by highlighting that maltreatment per se does not appear to be associated with impaired reward learning. Crucially, we also show for the first time that maltreatment and psychopathology do not interact to predict reward or punishment avoidance learning, which is suggestive of no latent vulnerability for that neurocognitive domain. Finally, psychopathology was associated with an impairment in punishment learning, which is in line with the current literature [27]. For instance, previous studies showed that subgroups with conduct problems exhibit reduced punishment learning compared to controls [27]. Some studies with adolescent samples indicate that this deficit may be specific to antisocial boys [55], but previous work on the same dataset did not replicate these findings [27].

In relation to our second aim, we found that sex interacted with maltreatment to predict performance in both emotion recognition and learning. Specifically, for emotion recognition, our study provides novel data indicating both similarities and differences in maltreated females and males. Indeed, we show for the first time that both maltreated sexes exhibit reduced recognition of fear, but that there are also sex differences such that females were impaired at recognizing happiness, while males were impaired at recognizing disgust. Since both maltreated males and females are at heightened risk of developing psychiatric disorders, similarities in their neurocognitive profile for fear recognition could reflect this. Interestingly, however, despite evidence that sex impacts both the nature and severity of psychiatric outcome following maltreatment, our supplementary analyses showed that our sample did not show sex differences in internalizing and externalizing psychopathology subtypes following maltreatment (Supplement 19).

The differences in recognizing happiness and disgust are also novel and suggest sex-dependent associations with maltreatment. Those findings are consistent with the literature suggesting that females and males respond to stress differently [29, 30] but here we show that sex differences in the neurocognitive profiles can be observed in maltreated youth specifically. Similarly, for emotion learning, our findings are novel and demonstrate a diametrically different profile across the sexes such that maltreated females made significantly more commission errors than non-maltreated females, whereas, surprisingly, maltreated males made less commission errors than non-maltreated males. Two previous studies using a similar task found lower punishment avoidance learning (i.e., lower correct rejection of punishment stimuli) in relation to maltreatment in boys [56] and to cumulative adverse experience in a mixed sex sample [23] while controlling for sex effects. Our findings clearly indicate that sex effects should be considered in future work investigating the association between maltreatment and emotion learning.

The strengths of our work can be divided into two categories. First, we included a large mixed-sex sample of youth who were comprehensively assessed using standardized measures for both maltreatment and psychopathology. Second, our unique design enabled us to clarify the main and interactive effects of maltreatment and psychopathology on emotion recognition and learning and test for sex effects. These findings, however, should be interpreted considering some limitations. First, by separating maltreatment from psychopathology, we inevitably obtained unequally sized groups. Specifically, the group with maltreatment and low psychopathology (i.e., resilient) was considerably smaller compared to the control and high psychopathology groups. However, the size of that group is comparable to resilient groups in other studies [57], where it is systematically reported that high functioning across time following maltreatment is as rare as 1.5%. Second, our effect sizes were rather small, which highlight substantial heterogeneity both within maltreatment and psychopathology. Third, the cross-sectional design precludes any causal inferences regarding temporal relation between maltreatment, psychopathology, and emotion recognition/learning and the extent to which emotion recognition performance in the resilient group reflects a true latent vulnerability for future psychopathology. Fourth, we assessed maltreatment based on interviews with parents/caregivers, who may not have been fully honest in reporting about maltreatment for social desirability reasons or may have been unaware that their child was abused. Fifth, our maltreatment measure (i.e., the CBE) does not distinguish between different subtypes of abuse (e.g., physical, or sexual), or the specific aspects of onset, severity, or chronicity of maltreatment. As such, we were not able to investigate their respective influences on emotion recognition and learning. However, these subtypes are often characterized by high co-occurrence, making it difficult to study them separately [58]. Relatedly, while we did not distinguish between different forms of psychopathology (e.g., internalizing vs. externalizing) in our main analyses, we acknowledge that these may be associated with different emotion recognition profiles [21, 59]. Following a reviewer’s suggestion, these post hoc analyses can be found in Supplementary 16. Crucially, however, neither internalizing nor externalizing symptoms scores interacted with any other variables to predict emotion recognition nor emotion learning. Furthermore, our study design treating psychopathology as one category fits with the literature showing that mental disorders are better characterized by a general psychopathology dimension (i.e., *p* factor) [61]*.* Finally, it is important to mention that the FemNAT-CD study was aimed primarily at investigating sex differences in CD, meaning that all the youth in the psychopathology groups had CD. However, many of these youth were characterized by other forms of comorbidity, such as PTSD (much higher prevalence in the maltreatment group with high psychopathology than in the group with high psychopathology, but no maltreatment), ADHD or MDD (See Supplement 21). Moreover, recent research investigating CD [60] in the same dataset showed that only 23% of youth with CD show impairments in emotion recognition.

While considerable evidence points to the detrimental effects of maltreatment on children’s emotional development, the inconsistencies in findings regarding emotion recognition and learning make it difficult to draw conclusions about their associations with maltreatment. Our study focused on disentangling the potential confounding effects of psychopathology from the effects of maltreatment on two domains of emotion processing—emotion recognition and learning as well as clarifying if sex moderates those associations. For emotion recognition, we found that (i) maltreated youth exhibited reduced recognition of fear, and (ii) that when maltreatment was high, but psychopathology was low, further deficits were observed in recognizing fear, happiness and disgust. These findings supported the latent vulnerability hypothesis for emotion recognition. For emotion learning, no evidence of altered reward or punishment learning, and no latent vulnerability was found in the maltreated groups. We also showed that maltreatment in females was associated with a lower recognition of fear and happiness, whereas maltreatment in males was associated with lower recognition of fear and disgust. Finally, for emotion learning, we showed that maltreated females were poorer at learning from punishment, compared to non-maltreated females, whereas maltreated males showed a reverse pattern (maltreated males made less commission errors than non-maltreated males). Should our findings be replicated in prospective longitudinal studies, they have the potential to clarify if the emotion recognition findings reflect true latent vulnerability and if the reported sex effects translate into males and females developing different forms of psychopathology long-term. They may also prove useful in informing interventions and therapies that target these domains, which have been associated with quality of friendships [8], peer and social skills [62] in adulthood.

### Supplementary Information

Below is the link to the electronic supplementary material.

Supplementary file1 (PDF 2101 KB)

## Data Availability

Data are available upon request from the FemNAT committee.
