# Supplementary material for: Emotion processing in maltreated boys and girls: Evidence for latent vulnerability
Source: Eur Child Adolesc Psychiatry. 2023 Feb 4;32(12):2523–36. doi: 10.1007/s00787-022-02132-1 (PMC10682268; doi:10.1007/s00787-022-02132-1)
Supplement: Supplementary file 1 — Supplementary file1 (PDF 2101 KB) [file 787_2022_2132_MOESM1_ESM.pdf]

Supplementary material for "Emotion processing in maltreated boys and girls. A latent vulnerability approach" by Diaconu et al.

Table of Contents

Supplementary material for "Emotion processing in maltreated boys and girls. A latent vulnerability approach" by Diaconu et al. 1

Supplement 1: Recruitment and Distribution of Participants per Group and Sex by Site 2

Supplement 2: Diagram of Participants Inclusion Criteria in the Two Studies 3

Supplement 3 : Participants' Demographic Characteristics and Psychopathology Subscales Scores. The Passive Avoidance Sample. 4

Supplement 4: Informed Consent 4

Supplement 5: Ethical Approval 5

Supplement 6: Data Collection Procedure 5

Supplement 7: Children's Bad Experiences (CBE) interview protocol 6

Supplement 8: Schedule for Affective Disorders and Schizophrenia for School-Age Children: Present and Lifetime Version (K-SADS-PL) 6

Supplement 9: Inter-Rater Reliability 7

Supplement 10: Imputation of Missing Data 7

Supplement 11: Task Randomization 8

Supplement 12: Socioeconomic Status (SES) 9

Supplement 13: ER and EL Results by largest data collection sites 9

Supplement 14: Pubertal Category and Age effects on Emotion Recognition and Learning 11

Supplement 15: Age by Emotion Interactions 13

Supplement 16: Emotion Recognition Results - Female sample 16

Supplement 17: The Passive Avoidance Learning Task. Results by Block 17

Supplement 18a: Confusability Matrix of Emotion Eesponses by Emotion Depicted by Groups ... 19

Supplement 18b: Percentages of Emotion Responses for Each Emotion Depicted by Group 19

Supplement 18c: Bayes Factors (BF_{10}) for the Confusability Matrix Data. Comparisons Between Control vs. Resilient and PT vs. MT +PT Groups. 21

Supplement 19: Sex Differences in Psychopathology Subtypes Following Maltreatment 22

Supplement 20. ER Analysis by Internalizing and Externalizing Subtypes of Psychopathology ... 23

Supplement 21: Participants' Comorbidities in the Psychopathology Groups 24

Supplement 1: Recruitment and Distribution of Participants per Group and Sex by Site

Recruitment took place between January 2014 and February 2018. In total, 1827 youths participated, including 880 with CD (61% females) and 947 TD youths (65% females). Data collection included questionnaires, behavioural, neuro-physiological and genetic measures, and structural and functional MRI data. Participants were recruited at 12 sites across Europe (Table 1). Sources included local clinics, mental health services, youth offending services, mainstream and special schools, youth clubs, other community services, outreach events and word of mouth.

TABLE 1. Distribution of participants per Group and Sex by Site

**Site**	**Total** *N*	**Control$_f$** *n*	**Control$_m$** *n*	**Resilient$_f$** *n*	**Resilient$_m$** *n*	**PT$_f$** *n*	**PT$_m$** *n*	**MT+PT$_f$** *n*	**MT+ PT$_m$** *n*
Aachen	251	65	57	8	4	37	32	25	23
Frankfurt	194	77	54	3	4	11	18	14	13
Birmingham	92	46	31	3	0	2	6	1	3
Athens	84	44	16	3	0	8	6	5	2
Bilbao	66	32	10	2	0	13	2	6	3
Southampton	39	18	16	1	0	0	4	0	0
Barcelona	33	13	0	0	0	10	4	4	1
Basel	29	22	0	2	0	4	0	1	0
Szeged	27	10	3	0	0	7	2	4	1
Amsterdam	12	0	0	0	0	8	0	4	0
Dublin	2	1	1	0	0	0	0	0	0
	**828**	**328**	**188**	**22**	**8**	**100**	**72**	**64**	**46**

Note: $_{f/m}$=female/male; Control = No Psychopathology, No Maltreatment; Resilient = Probable/Definite Maltreatment only; PT= High Psychopathology only; MT+PT=Probable/Definite Maltreatment, and High Psychopathology.

Supplement 2: Diagram of Participants Inclusion Criteria in the Two Studies

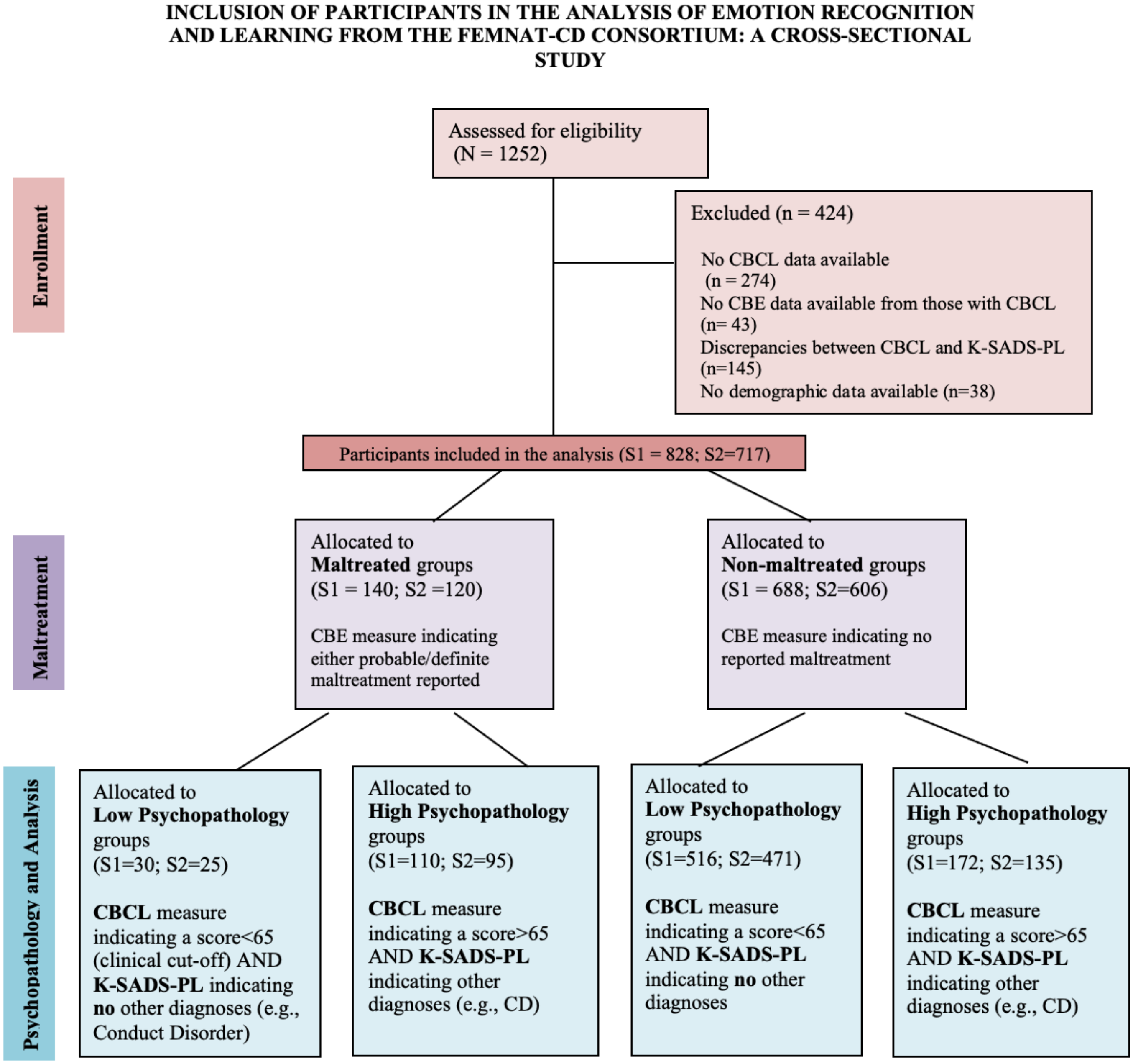

Supplement 3 : Participants' Demographic Characteristics and Psychopathology Subscales Scores. The Passive Avoidance Sample.

	Control	Resilient	High Psychopathology	Psychopathology + Maltreatment	Group effects
**Sample** **Study 2**	**N=471[2]**	**N=25[2]**	**N=135[2]**	**N=95[2]**	
**Age, M (SD)**	13.71 (2.5)	14.2 (2.4)	14.2 (2.4)	14.03(2.4)	0.9
**Females (in %)**	66.1	76.6	58.8	50.6	5.8
**Estimated IQ, M (SD)**	107.1 (10.7) [a]	103.1 (10.5) [a]	96.4 (10.2) [b]	96.7 (13.8) [b]	12.7***
**Estimated Verbal IQ, M (SD)**	105.6 (13.2) [a]	102.6 (14.2) [a]	96.8 (14.6) [b]	93 (17.6) [b]	21.4***
**Estimated Performance IQ, M (SD)**	105 (13.1) [a]	102.6 (14.2) [a]	96.8 (14.6) [b]	102.1 (14.2) [a]	7.8***
**PDS (1=pre/early puberty; 2=mid/late/post puberty) (in %)**	2 – 82.4	2 - 80	2 – 81.1	2 – 84.6	0.1
**SES M (SD)**	0.4 (0.9) [a]	0 (1.1) [a]	-0.3 (1) [b]	-0.5 (1.1) [b]	21.8***
**CBCL Total t scores M (SD)**	48.2 (8.2) [a]	51.6 (6.9) [a]	75.5 (7.2) [b]	76.3 (6.1) [b]	442.6***
**CBCL Internalising Scale**	47.6 (7.9) [a]	50.6 (7.5) [a]	75.8 (6.2) [b]	76.6 (5.9) [b]	508.7***
**CBCL Externalising Scale**	49.8 (8.2) [a]	53.2 (6.9) [a]	69.3 (8.9) [b]	71.1 (9.0) [b]	198.2***
**CBCL Anxiety/Depression**	53.2 (4.8) [a]	53.6 (4.9) [a]	68.4 (10.3) [b]	71.2 (9.2) [b]	204.9***
**CBCL Withdrawal**	53.6 (5.2) [a]	55 (5.7) [a]	67.1 (9.8) [b]	68.8 (9.7) [b]	145.5***
**CBCL Somatic Complaints**	54.6 (5.8) [a]	58.2 (7) [a]	65.5 (10.7) [b]	65.5 (11.5) [b]	66.7***
**CBCL Social Problems**	52.5 (4.6) [a]	54.5 (4.1) [a]	69 (9.5) [b]	69.6 (9.4) [b]	233.2***
**CBCL Thought Problems**	52.2 (4.4) [a]	53.6 (5.1) [a]	67.8 (10.2) [b]	67.3 (10.9) [b]	176.5***
**CBCL Attention Problems**	52.7 (5.2) [a]	53.6 (4.1) [a]	72.7 (8.9) [b]	75 (10.5) [b]	333.2***
**CBCL Rule-breaking Behaviour**	52.3 (4.3) [a]	53.6 (4.2) [a]	74.9 (8.6) [b]	77.2 (7.6)	578.7***
**CBCL Aggressive Behaviour**	52.3 (3.8) [a]	53.5 (3.9) [a]	77.3 (8.6) [b]	77.5 (9.4) [b]	626.4***

Notes: Control = No Maltreatment, Low Psychopathology; Resilient= Probable/Definite Maltreatment, Low Psychopathology; High Psychopathology=No Maltreatment, High Psychopathology; Psychopathology + Maltreatment = High Psychopathology, Probable/Definite Maltreatment; SES = Socioeconomic Status (SES was computed based on parental income, education level and occupation); CD = Conduct Disorder (Diagnosis of CD was based on the Schedule for Affective Disorders and Schizophrenia for School-Age Children-Lifetime and Current versions (KSADS-L/C); PDS = Pubertal Developmental Status; CBCL = Child Behaviour Checklist; The CBCL scores for the Internalising scale were computed using the Anxiety/Depression, Withdrawal and Somatic Complaints subscales, whereas the scores for the Externalising scale were computed using the Rule-breaking and Aggressive Behaviour subscales; EMM = Estimated Marginal Mean; SEM = Standard Error of the Mean; Post-hoc tests are reported based on observed means, where groups marked with different letter differ significantly from each other at *p<.05, **p<.01 and ***p<.001.

Supplement 4: Informed Consent

A parent, guardian or other responsible adult gave written informed consent on behalf of minors (*i.e.*, those below 16 or 18 years depending on site of data collection). Minors gave written assent. Parents and guardians, and participants above the required age, gave written

Supplement 5: Ethical Approval

The FemNAT-CD study (www.femnat-cd.eu) was conducted in accordance with legal regulations outlined by the European Union, national legislation and the Declaration of Helsinki. Study protocols were approved by local ethical committees at each site prior to data collection: RWTH Aachen University Hospital (EK027/14) for the Aachen site (site 1), the Ethics Commission Northwest and Central Switzerland (EKNZ: 336/13) for the Basel site (site 2), and the University Ethics Committee (ERGO Number: 18970) and the National Health Service Research Ethics Committee (NRES Committee West Midlands, Edgbaston; REC Reference 13/WM/0483) for the two UK sites (Birmingham (site 3) and Southampton (site 4)).

Supplement 6: Data Collection Procedure

Referrals to the project were initially screened by telephone to assess suitability. Participants judged likely to be eligible, and their parents or guardians, were then invited to a more comprehensive screening session, either at the local site (*i.e.*, university or associated clinic), or at the participant's home. The first session generally lasted 1-3 hours. Informed consent was provided at the beginning of the session. Participants and their parents/guardians were interviewed separately by trained researchers to assess for the presence of any mental disorders. An IQ test was also administered. Eligible participants then returned for a second session of approximately two hours, in which they completed questionnaire measures, provided saliva samples for genotyping, and completed a set of neurophysiological and neurocognitive assessments. Data on parenting and facial emotion recognition were usually collected during this session. Participants received a small financial reimbursement, or equivalent vouchers, after each session, as approved by the local ethics committees[1].

[1] Aachen: Ethik Kommission Medizinische Fakultät der Rheinisch Westfälischen Technischen Hochschule Aachen (EK027/14). Amsterdam: Medisch Etische Toetsingscommissie (2014.188). Athens: Election Committee of the First Department of Psychiatry, Eginition University Hospital (641/9.11.2015). Barcelona: Child and Adolescent Mental Health - University Hospital Mutua Terrassa (acta 12/13). Basel: Ethik Kommission Nordwest- und Zentralschweiz (EKNZ 336/13). Bilbao: Hospital del Basurto. Birmingham and

Supplement 7: Children's Bad Experiences (CBE) interview protocol

The caregivers responded to standardized probe questions aimed at investigating whether the child/adolescent had experienced i) harm on purpose, ii) harm in the course of discipline, iii) sexual harm, or iv) harm that led to the involvement of an agency (e.g., social services). Examples of probe questions included "Do you remember any time when the child was disciplined severely enough that he or she may have been hurt?" or "Has there been any other situation where your child has been harmed by someone (not accidents)? This could include sexual harm, such as flashing". Following each discussion, interviewers rated privately the probability that the child had been severely harmed. For instance, in the case of physical harm, using a criterion of intentional strikes to the child by an adult that left visible marks for more than 24 hours or that required medical attention, a score of 0 was assigned if maltreatment had definitely not or probably not occurred, and a score of 1 was assigned if maltreatment had probably occurred, definitely occurred, or if authorities had been involved. (Lansford et al., 2002). Each rater's judgement (i.e., no/probable/definite maltreatment) was compared with scores from a neutral rater and agreement between the independent raters was reported as 90% (($\kappa = 0.56$) in previous studies (Dodge, Pettit, Bates, & Valente, 1995) and in ours.

Supplement 8: Schedule for Affective Disorders and Schizophrenia for School-Age Children: Present and Lifetime Version (K-SADS-PL)

The K-SADS-PL (Kaufman *et al.*, 1997) is a semi-structured diagnostic interview used to assess current and past psychopathology in children and adolescents. The interview was

Southampton: University Ethics Committee and National Health Service Research Ethics Committee (NRES Committee West Midlands, Edgbaston; REC reference 3/WM/0483). Dublin: SJH/AMNCH Research Ethics Committee (2014/04/Chairman (3)). Frankfurt: Ethik Kommission Medizinische Fakultät Goethe Universität Frankfurt am Main (445/13). Szeged (Hungary): Egészségügyi Tudományos Tanács Humán Reprodukciós Bizottság (CSR/039/00392-3/2014).

administered separately to participants and parents (or another responsible adult informant) by trained researchers, and combined parent and child summary ratings of all symptoms (past, present and lifetime) were then generated. Where assessors gave discrepant ratings for a symptom, they discussed all available information until an agreement was reached for the summary rating. Except for CD, ODD, and ADHD, where DSM-5 criteria were used, all diagnoses were generated based on the DSM-IV-TR diagnostic criteria, which were current at the outset of the project (APA, 2000). Inter-rater reliability for current CD diagnoses was high (94.7% agreement across raters, Cohen's kappa=0.91).

Supplement 9: Inter-Rater Reliability

Inter-rater reliability (IRR) values were calculated for comorbid disorders, notably ADHD), MDD, ODD and GAD (Cohen's kappas >= 0.84 and agreement across trained raters >= 92% for all diagnostic categories). The severity of CD and comorbid disorders was defined based on the number of symptoms endorsed across informants (i.e. a symptom was considered present if endorsed by either informant). The inter-rater reliability was conducted on a subsample of participants (n=75) from across all of the FemNAT-CD research sites. For ethical reasons, at the UK sites two interviewers attended the sames interviews but coded the interviews seperately; at all other sites, interviews were videotaped and scored by different raters.

Supplement 10: Imputation of Missing Data

Missing values of the PDS score were imputed based on the whole FemNAT-CD sample. It has been shown that missing data in a multi-item instrument is best handled by imputation at the item level (Eekhout, de Vet, Twisk, Brand, de Boer, & Heymans, 2014). Thus, missing values of the single items were imputed first and the scores were calculated based on the imputed items. The imputation was done in SAS® version 9.4 using the procedure PROC MI. Imputation by fully conditional specification (FCS) is used, which offers a flexible method to specify the multivariate

imputation model for arbitrary missing patterns including both categorical and continuous variables (Liu & De, 2015). As the items are measured at an ordinal level, the logistic regression method is specified in the FCS statement. For imputation diagnostics, distribution of the observed and imputed items and scores were checked. The imputation of the PDS items was done separately in males and in females because of sex specific items: item 2 (females and males) and items 4, 5a of the form for females or items 4, 5 of the form for males were imputed respectively. The following variables were included in the imputation model: sex specific items of the PDS as mentioned above and the two remaining PDS items (items 1 and 3), age at PDS and age at informed consent, to impute age at PDS if missing, weight, case/control status, site, and migration status. Imputation for the remaining measures was conducted separately, following the same procedure as above. The following variables were included in the imputation model: all items of the respective questionnaire, age, IQ, group (case/control), sex (male/female), site, comorbidities (PTSD, ADHD, ODD, depression, anxiety), and items of other questionnaires if correlated with at least one of the items with ≥0.4. For imputation diagnostics, distribution of the observed and imputed items and scores were checked.

Supplement 11: Task Randomization

Participants completed a neuropsychological test battery comprised of the Emotional Hexagon Task, the Passive Avoidance Learning Task and the Emotional Go/No Go Task (not included in this study). Each clinical site was provided with a "Permutation List" for the computerized test battery including the different task orders. Task order was randomized separately for age (i.e., 9-12 yrs., 13-15 yrs., and 16-18 yrs.), group (i.e., Psychopathology/Conduct Disorder (CD) and Typically Developed Controls (TDC)) and gender (i.e., female, male). Permutation number of task order (1 to 6) was noted and entered into the online database. Tasks were randomized according to the following scheme:"

Female cases, age 9-12 years Page No[i]. |____|

Study-ID	Subject-ID for Computer tests[ii]	Permutation No.	Test/Task 1	Test/Task 2	Test/Task 3	First Video Clip
01-|_|_|_|_|	|_|_|_|_|_|	1	Hexagon	Go/Nogo	Avoidance	bear
01-|_|_|_|_|	|_|_|_|_|_|	2	Hexagon	Avoidance	Go/Nogo	champ
01-|_|_|_|_|	|_|_|_|_|_|	3	Go/Nogo	Hexagon	Avoidance	bear
01-|_|_|_|_|	|_|_|_|_|_|	4	Go/Nogo	Avoidance	Hexagon	champ
01-|_|_|_|_|	|_|_|_|_|_|	5	Avoidance	Hexagon	Go/Nogo	bear
01-|_|_|_|_|	|_|_|_|_|_|	6	Avoidance	Go/Nogo	Hexagon	champ
6-digit Study-ID	5-digit Subject-ID for Neuropsych. Testing		<--------- Randomized Task order --------->			

FIGURE A. Example of Permutation List with Study-ID, Subject-ID and the six possible task orders

Supplement 12: Socioeconomic Status (SES)

Standardized factor scores for socioeconomic status (SES) were computed (mean = 0, SD = 1) based on parental income, education and occupation. Assessments were based on the International Standard Classification of Occupations (ISCO-08; International Labour Organisation, 2012) and the International Classification of Education (ISCED; UNESCO, 2015). Due to potential economic variation on the country level, SES was centered and scaled within each country, in order to obtain an indicator of relative socioeconomic position. Reliability (internal consistency) of the composite SES score was acceptable (Cronbach's Alpha = .74). The reported SES values are comparable with previous large samples derived from the FemNAT-CD consortium.

Supplement 13: ER and EL Results by largest data collection sites

The FemNAT-CD is a multi-site study and to account for potential confounds related to participants being tested at different European locations, we have re-run the analyses by only including the sites with the biggest numbers of participants (i.e., Germany and UK, N= 576). For emotion recognition, the analysis of covariance showed main effects of psychopathology, sex, emotion and morph. There were also significant interactions between emotion and sex, and emotion, psychopathology and maltreatment. These results are in line with the ones

reported in the main manuscript, where data from all 11 sites were analyzed. However, there were also some discrepancies, in that the emotion by maltreatment interaction was no longer significant (*p*=.072), and neither the emotion by sex by maltreatment (.055). For emotion learning, re-running the analysis with these two sites only, resulted in the same pattern of results. For avoidance errors, we found main effects of psychopathology, sex and block, two-way interactions between sex and maltreatment and sex and psychopathology, and the same sex by maltreatment by block, and sex by psychopathology by block (Table 2).

TABLE 2. Main and Interactive Effects of CM, Psychopathology and Sex on Emotion Recognition and Emotion Learning (UK + Germany sites).

Emotion Recognition

Main effects	*F*	*df*	*p*	η_p^2	*Post-hoc comparisons*
**Maltreatment**	1.24	1, 562	.266	.002	-
**Psychopathology**	6.5	1, 562	.011	.011	-
**Sex**	17.64	1, 562	*p*<.001	.030	-
**Emotion**	19.64	5, 2810	*p*<.001	.034	Happiness > Surprise = Sadness > Anger > Fear > Disgust (All emotions differed significantly at p<.001 level, except from Surprise vs. Sadness (*p*>.05)
**Morph**	7.98	1, 562	.005	.014	-
2-Way Interactions					
**Emotion by Maltreatment**	1.86	5, 562	.172	.000	-
3-Way Interactions					
**Emotion by Maltreatment by Psychopathology**	5.5	5, 562	.019	.010	Low Psychopathology:

					**Happiness, Fear, Disgust**:<br><br>Maltreated < Non-Maltreated<br><br>Anger, Surprise, Sadness: n.s.<br><br>High Psychopathology: n.s.
**Emotion by Sex by Maltreatment**	2.16	5, 4040	.055	.004	-

Emotion Learning

Main effects	***F***	***df***	***p***	η_p^2	***Post-hoc comparisons***
**Psychopathology**	8.17	1, 498	.004	.016	-
**Sex**	5.53	1, 498	.019	.011	-
2-Way Interactions					
**Sex by Maltreatment**	5.58	1, 498	.018	.011	Maltreated Females > Maltreated Males<br><br>Non-Maltreated: n.s.
**Sex by Psychopathology**	4.09	1, 498	.043	.008	Low Psychopathology: Females > Males<br><br>High Psychopathology: n.s.

Notes: Covariates evaluated in the model were SES = 0.125, mean-centered IQ=1.245, and pubertal status (1 = pre/early puberty; 2 = mid/late/post puberty). The adjustment for multiple comparisons was obtained using the Bonferroni correction.

Supplement 14: Pubertal Category and Age effects on Emotion Recognition and Learning

TABLE 3. Pubertal category effects in the Emotion Recognition and Emotion Learning Analyses

Emotion Recognition

Main effects	*F*	*df*	*p*	η_p^2	*Post-hoc comparisons*
**Pubertal category**	35.14	1, 808	*p*<.001	.042	Post >Pre

Emotion Learning

Main effects	*F*	*df*	*p*	η_p^2	*Post-hoc comparisons*
**Pubertal category**	8.43	1, 707	.004	.012	Post>Pre

Notes: Covariates evaluated in the model were SES = 0.125, mean-centered IQ=1.245, and pubertal status (1 = pre/early puberty; 2 = mid/late/post puberty). The adjustment for multiple comparisons was obtained using the Bonferroni correction.

TABLE 4. Main and Interactive Effects of Maltreatment, Psychopathology and Sex on Emotion Recognition and Learning *with age replacing pubertal category as a covariate*

Emotion Recognition

Main effects	*F*	*df*	*p*	η_p^2	*Post-hoc comparisons*
**Maltreatment**	5.31	1, 817	.021	.006	MT < nMT
**Psychopathology**	3.84	1, 817	.05	.005	High < Low
**Sex**	26.70	1, 817	*p*<.001	.032	Females > Males
Covariates					
**Age**	119.87	1,817	*p*<.001	.128	-
**SES**	23.73	1,817	*p*<.001	.028	-
**IQ**	30.35	1,817	*p*<.001	.036	-
2-Way Interactions					
**Emotion by Maltreatment**	2.52	5, 4085	.027	.002	**Fear:** MT < nMT<br>Anger, Happiness, Surprise, Sadness, Disgust: n.s.
**Emotion by Age**	6.17	5, 4085	*p*<.001	.008	-
**Emotion by SES**	4.24	5, 4085	*p*<.001	.005	-

**Emotion by IQ**	2.4	5, 4085	.035	.003	-
3-Way Interactions					
**Emotion by Maltreatment by Psychopathology**	2.22	5, 4085	.049	.010	Low Psychopathology: n.s.<br>High Psychopathology: n.s.
**Emotion by Sex by Maltreatment**	3.01	5, 4085	.01	.002	MT comparisons: n.s.<br><br>Between Sex comparisons:<br><br>No Maltreatment: **Females > Males (Fear, Disgust)**<br><br>Maltreatment: **Females > Males (Disgust)**

Emotion Learning

Main effects	*F*	*df*	*p*	η_p^2	*Post-hoc comparisons*
**Psychopathology**	7.89	1, 715	.005	.011	High < Low
**Sex**	4.69	1, 715	.031	.007	Females > Males
2-Way Interactions					
**Sex by Maltreatment**	5.17	1, 715	.023	.007	n.s.
**Sex by Psychopathology**	4.5	1, 715	.034	.006	Males:<br>High Psychopathology> Low Psychopathology<br>Females: n.s.

Notes: Covariates evaluated in the model were SES = 0.125, mean-centered IQ=1.245, and pubertal status (1 = pre/early puberty; 2 = mid/late/post puberty). The adjustment for multiple comparisons was obtained using the Bonferroni correction.

Supplement 15: Age by Emotion Interactions

We have further explored the significant interaction between age and emotion. Consistent with previous literature, recognition accuracy improved with age for all emotions except happiness. For descriptive purposes, we have now included graphs showing the % recognition accuracy for each of the six emotions, in each of the ten age groups (9y old to 18 y old). For ease of interpretation, we have divided these age groups into 2 age categories (i.e., Category 1 = 9 to 13 years old; Category 2 = 14 to 18 years old) and ran a 2 (age category) by 6 (emotions) analysis of variance. This analysis highlighted the same pattern of results, in that there was a main effect of age category ($F(1,826)=69.82$, $p<.001$, $\eta_p^2=.078$; 2>1), which also significantly interacted with all emotions, except happiness ($F(5,4130)=5.62$, $p<.001$, $\eta_p^2=.007$).

FIGURE B. Emotion by age groups interaction in the Emotion Hexagon Task.

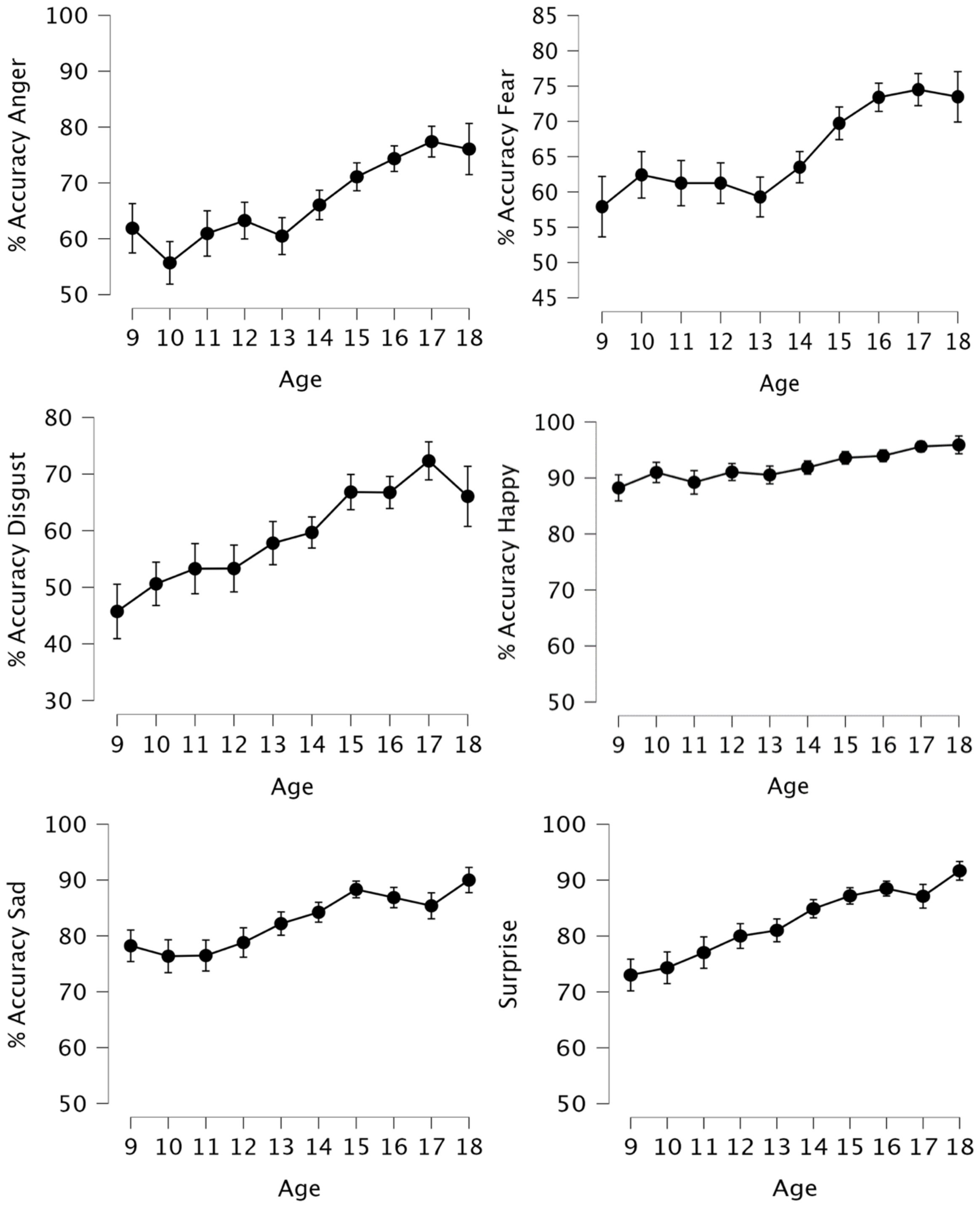

FIGURE C. Emotion by age category interaction in the Emotion Hexagon Task.

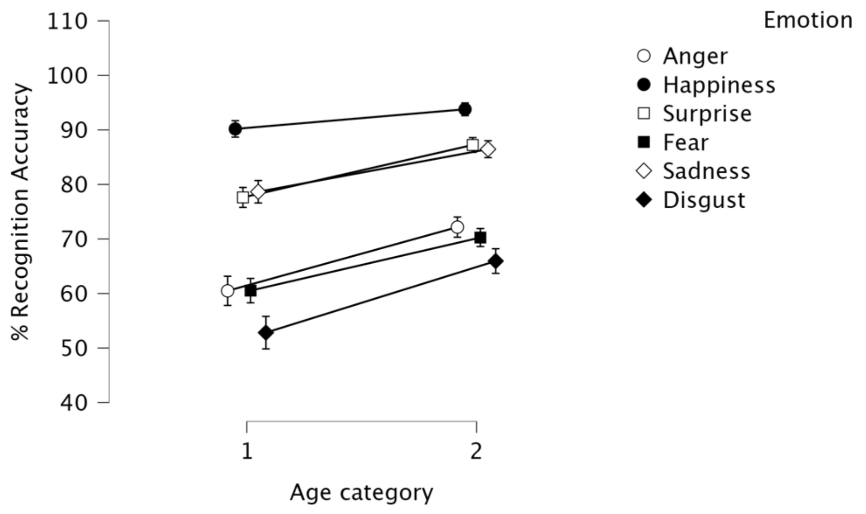

Supplement 16: Emotion Recognition Results - Female sample

TABLE 5. Main and Interactive Effects of CM and Psychopathology on Emotion Recognition. ***Female sample only.***

			**Emotion Recognition**		
Main effects	***F***	***df***	***p***	η_p^2	***Post-hoc comparisons***
**Maltreatment**	1.62	1, 507	.204	.003	nMT > MT
**Psychopathology**	8.35	1, 507	.004	.016	Low > High
Covariates					
**Pubertal category**	24.98	1,507	*p*<.001	.047	-
**SES**	10.01	1,507	.002	.028	-
**IQ**	9.1	1,507	.003	.018	-
2-Way Interactions					
**Emotion by Maltreatment**	2.28	5, 2535	.044	.004	n.s.
3-Way Interactions					
**Emotion by Maltreatment by Psychopathology**	0.87	5, 2535	>.05	.002	-

Notes: Covariates evaluated in the model were SES = 0.125, mean-centered IQ=1.245, and pubertal status (1 = pre/early puberty; 2 = mid/late/post puberty). The adjustment for multiple comparisons was obtained using the Bonferroni correction.

FIGURE D. Interactions between emotion, maltreatment, and psychopathology in the female sample

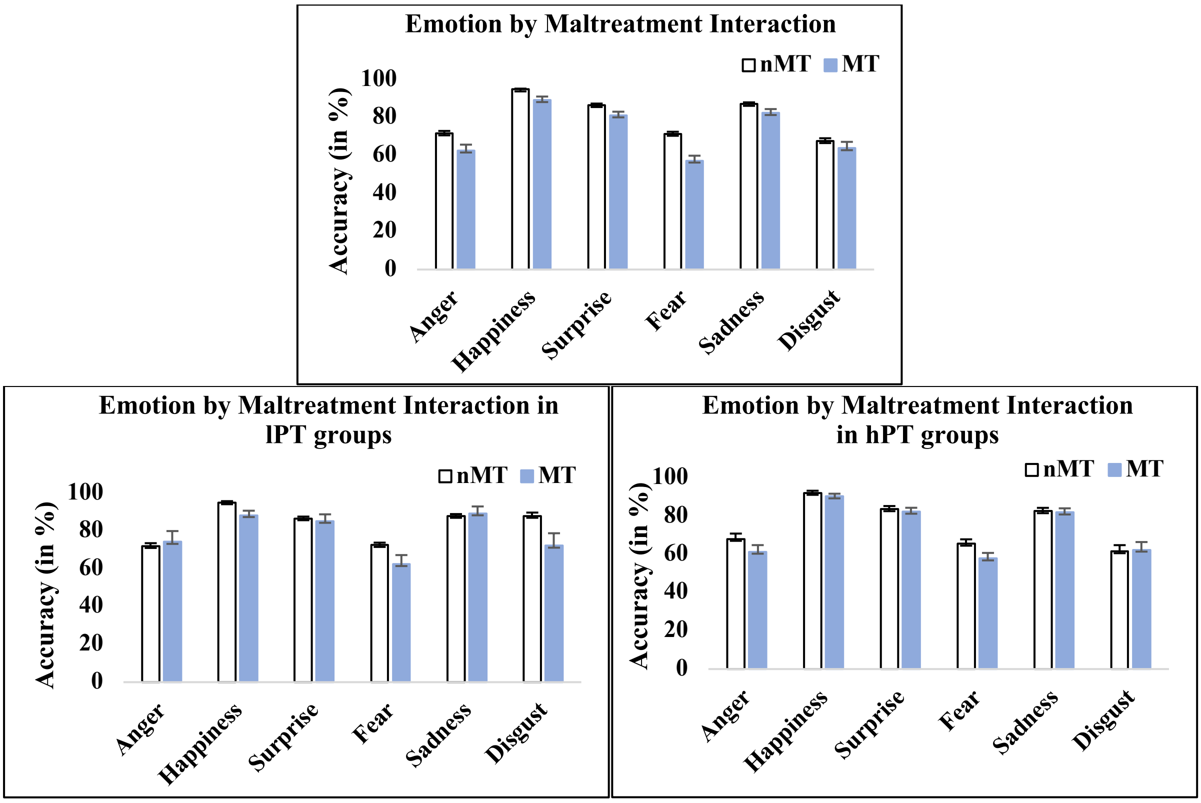

Supplement 17: The Passive Avoidance Learning Task. Results by Block

In the passive Avoidance Learning task, participants completed 10 blocks, the first one being for practice and therefore not included in the analysis. For simplification reasons, we have left these results out of the manuscript, reporting them here instead. The analysis indicated a significant three-way interaction between block, sex and maltreatment (*F*(8,5656)=4.38, *p*<.001, η_p^2=.006) and between block, sex and psychopathology ((*F*(8,5656)=2.93, *p*=.003, η_p^2=.004). For the former, pairwise comparisons indicated that maltreated females made more avoidance errors compared to maltreated males during blocks 3,6,7, and 8. For the latter, low

psychopathology females made more avoidance errors than low psychopathology males during the same blocks (Figure E).

FIGURE E. Interactive Effects of Maltreatment, Psychopathology, and Sex on Avoidance Errors. Results by Block.

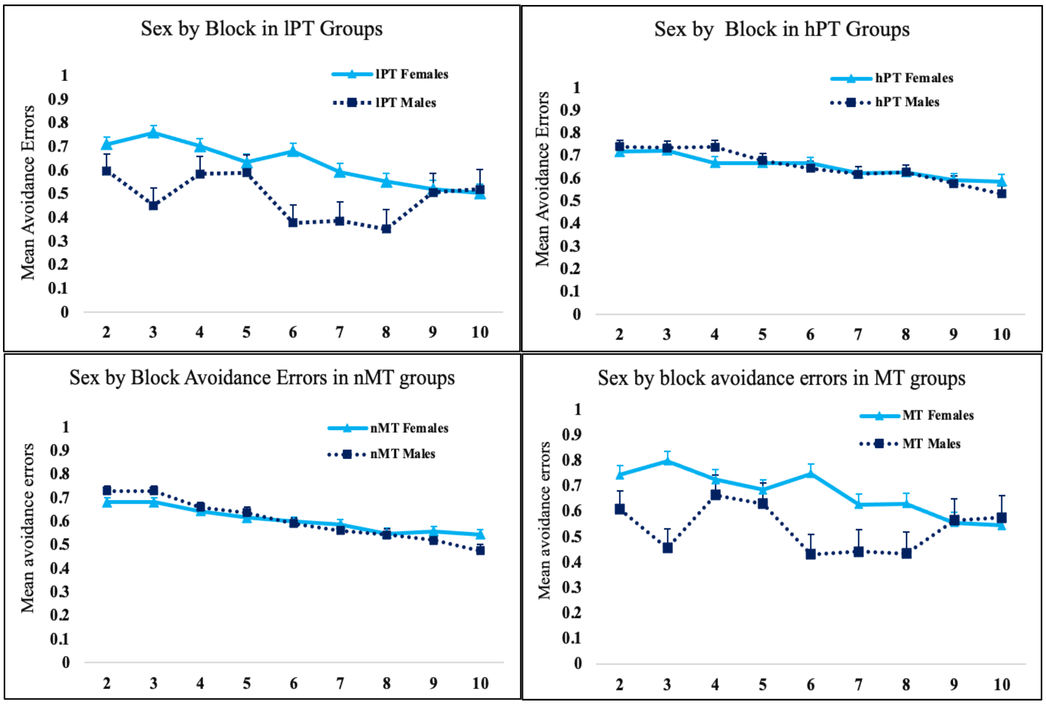

Supplement 18a: Confusability Matrix of Emotion Eesponses by Emotion Depicted by Groups

	Emotion identified					
**Emotion depicted**	***Anger***	***Fear***	***Disgust***	***Happiness***	***Sadness***	***Surprise***
**Control**						
Anger	-	3.90 (6.79)	16.23 (21.16)	0.55 (1.97)	1.42 (3.68)	7.03 (10.74)
Fear	1.26 (3.17)	-	6.32 (11.40)	0.66 (2.60)	2.64 (5.53)	16.84 (15.51)
Disgust	19.14 (26.28)	1.78 (4.52)	-	0.62 (2.46)	11.02 (12.27)	1.20 (4.59)
Happiness	0.66 (2.24)	0.80 (2.34)	0.82 (2.32)	-	0.93 (2.91)	1.73 (4.30)
Sadness	1.97 (1.97)	3.40 (6.26)	5.96 (10.72)	0.68 (2.28)	-	1.23 (3.64)
Surprise	0.84 (2.55)	7.42 (11)	1.80 (5.35)	2.57 (4.79)	0.90 (2.75)	-
**PT**						
Anger	-	6.71 (12.58)	15.98 (19.35)	2.44 (6.36)	2.76 (5.54)	10.20 (12.23)
Fear	2.90 (5.56)	-	11.36 (13.85)	1.83 (4.74)	3.83 (6.54)	22.67 (18.16)
Disgust	22.26 (26.84)	3.69 (8.64)	-	1.91 (4.75)	13.43 (13.91)	2.64 (5.65)
Happiness	2.15 (4.46)	1.83 (4)	2.44 (5.42)	-	2.20 (4.23)	4.01 (6.41)
Sadness	3.77 (3.77)	5.98 (9.48)	8.83 (14.03)	1.80 (5.15)	-	4.01 (8.12)
Surprise	2.15 (4.08)	10.37 (15.19)	3.54 (7)	4.30 (7.15)	2.47 (4.91)	-
**Resilient**						
Anger	-	4.16 (7.77)	15.66 (18.83)	1.00 (2.42)	1.83 (5.54)	4.66 (6)
Fear	2.33 (4.68)	-	18.50 (23.38)	1.33 (3.45)	1.66 (6.54)	17.83 (14.54)
Disgust	25.00 (34.34)	3.16 (7.24)	-	0.83 (2.30)	13.33 (13.91)	1.66 (4.42)
Happiness	4.83 (15.83)	1.66 (3.30)	2.33 (4.86)	-	1.33 (4.23)	1.66 (3.03)
Sadness	1.33 (1.33)	3.50 (6.96)	6.50 (7.89)	1.16 (3.13)	-	1.33 (3.45)
Surprise	0.66 (1.72)	7.33 (8.38)	4.16 (7.20)	2.66 (3.65)	1.50 (4.91)	-
**MT + PT**						
Anger	-	5.31 (8.1)	21.13 (23.88)	2.36 (4.87)	2.72 (5.14)	10.50 (13.07)
Fear	3.68 (6.72)	-	12.95 (15.43)	2.22 (4.95)	4.50 (6.01)	22.54 (16.41)
Disgust	23.86 (27.55)	3.54 (5.55)	-	1.40 (4.01)	12.18 (11.81)	3.18 (5.93)
Happiness	2.04 (4.13)	1.86 (4.43)	2.50 (4.77)	-	2.50 (4.82)	2.81 (5.88)
Sadness	3.81 (3.81)	6.86 (7.77)	7.68 (11.65)	2.13 (5.13)	-	2.63 (5.36)
Surprise	2.86 (6.78)	8.31 (9.41)	5.13 (8.55)	3.45 (5.93)	2.50 (4.53)	-

Notes: This table shows means (and standard deviations) in % of participants' response (i.e., emotion identified) for each of the six emotions depicted; **Control** = No maltreatment, no psychopathology; **PT** = High Psychopathology, no Maltreatment; **Resilient** = Maltreatment, Low Psychopathology; MT+PT = Maltreatment, High Psychopathology.

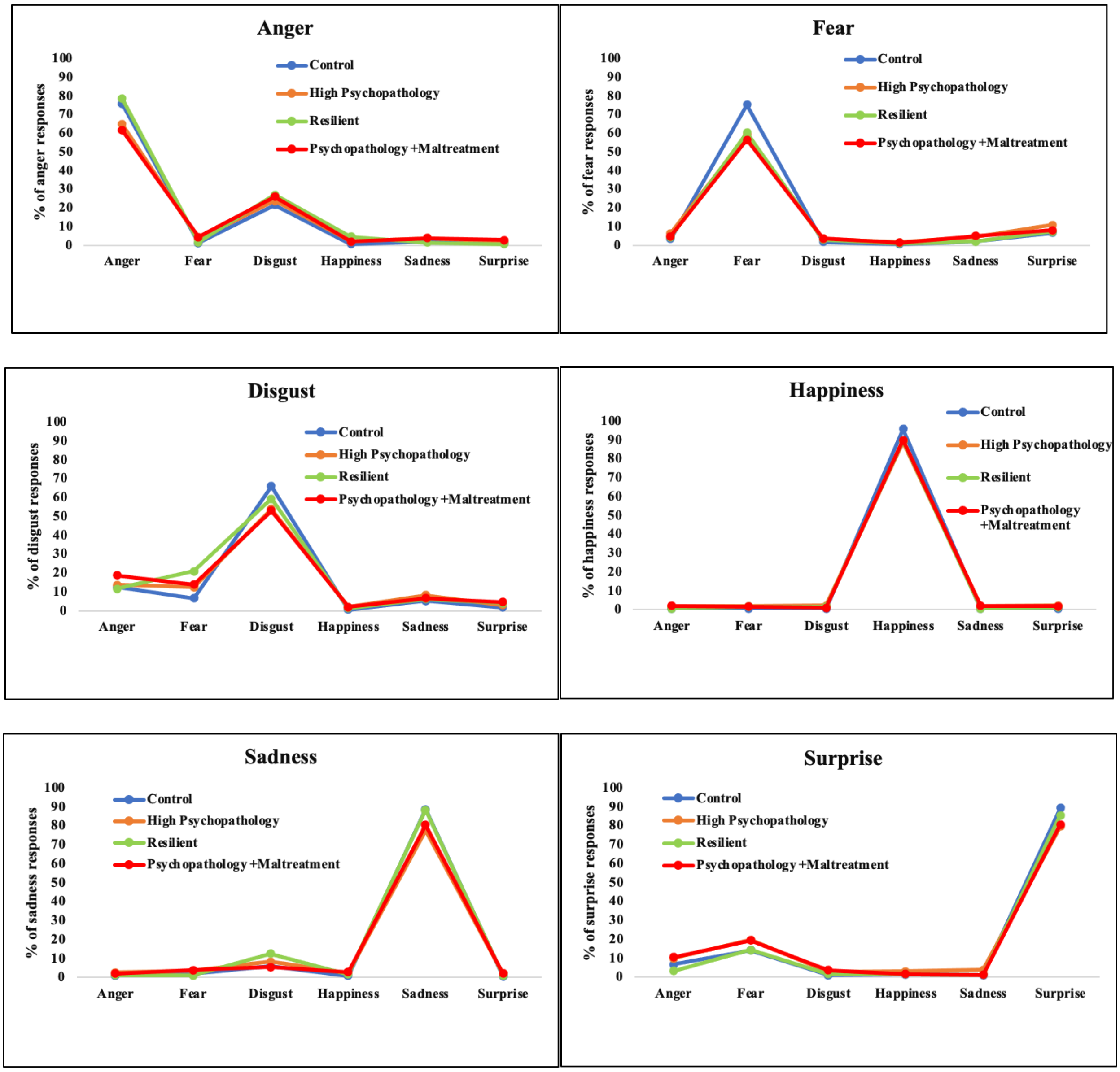
Anger
% of anger responses
Fear
% of fear responses
Disgust
% of disgust responses
Happiness
% of happiness responses
Sadness
% of sadness responses
Surprise
% of surprise responses
Control
High Psychopathology
Resilient
Psychopathology +Maltreatment
0
10
20
30
40
50
60
70
80
90
100
Anger
Fear
Disgust
Happiness
Sadness
Surprise

Supplement 18c: Bayes Factors (BF_{10}) for the Confusability Matrix Data. Comparisons Between Control vs. Resilient and PT vs. MT +PT Groups.

	Emotion identified					
**Emotion depicted**	***Anger***	***Fear***	***Disgust***	***Happiness***	***Sadness***	***Surprise***
**Control vs. Resilient**						
Anger	-	0.24	0.20	0.20	0.20	0.53
Fear	0.24	-	103521.47*	1.41	0.32	0.20
Disgust	0.29	0.32	-	1.55	2.27	0.24
Happiness	674986.71*	0.32	0.42	-	0.38	0.20
Sadness	0.22	0.20	0.22	0.20	-	0.20
Surprise	0.22	0.20	0.92	0.67	0.24	-
**PT vs. MT +PT**						
Anger	-	0.26	0.66	0.13	0.17	0.14
Fear	0.28	-	0.17	0.14	0.14	0.13
Disgust	0.15	0.13	-	0.53	0.36	0.25
Happiness	0.13	0.13	0.13	-	0.18	0.66
Sadness	0.13	0.15	0.18	0.13	-	1.18
Surprise	0.21	0.33	0.46	0.14	0.13	-

***Notes**:* This table shows BF_{10} factors for the comparisons of emotion responses between the control and maltreated group (i.e., resilient) and between the high psychopathology versus high psychopathology and maltreatment group, for each of the six emotions depicted.

Bayes Factor Analysis showed that the resilient group was different compared to the control group for responses of anger and disgust. Specifically, resilient youth showed more response bias towards anger when happiness was depicted, and more disgust responses when fear was depicted (Figure F).

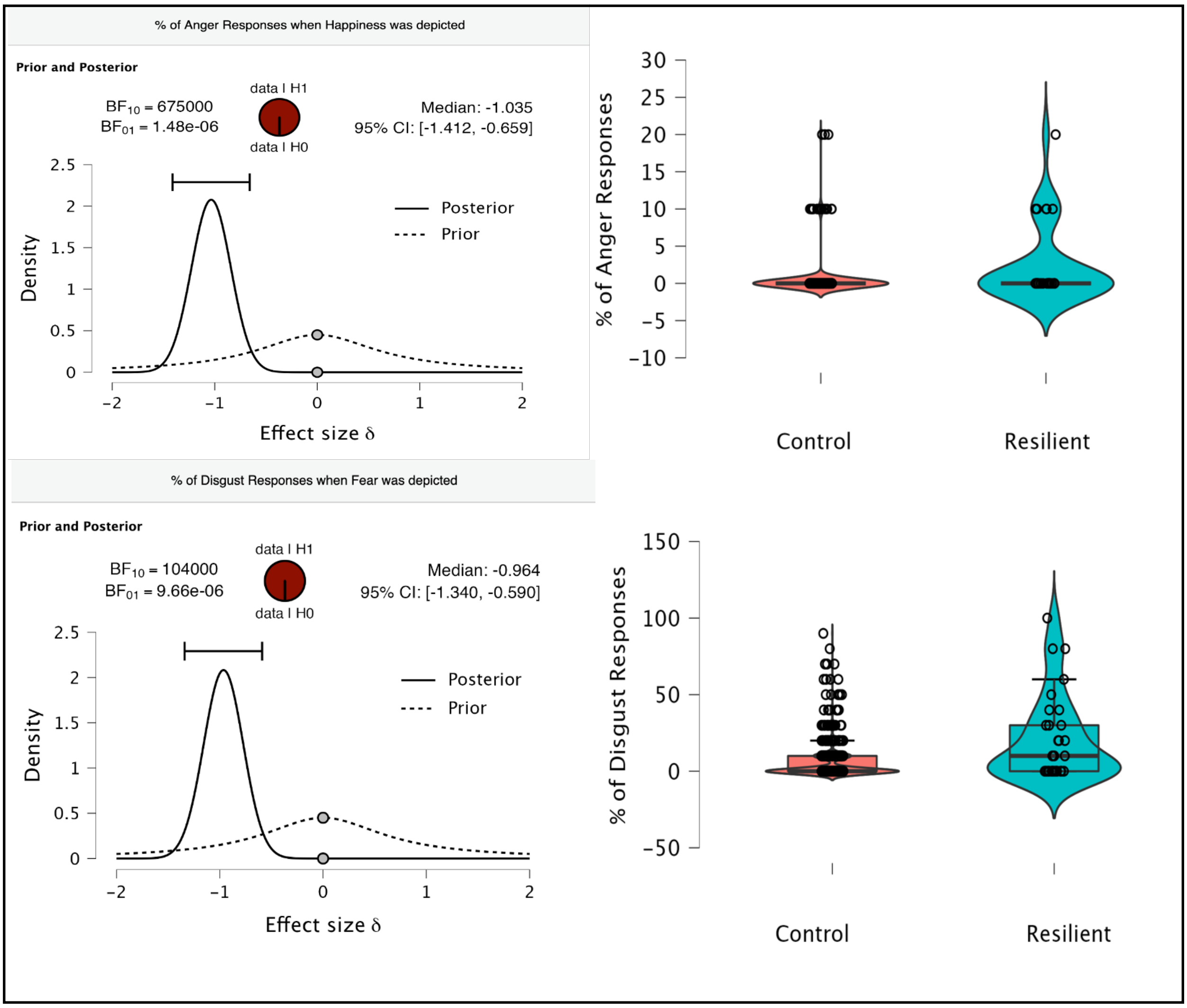

Notes: The panels on the right show mean % of anger and disgust responses between the control and resilient groups. Errors bars show SEM.

Supplement 19: Sex Differences in Psychopathology Subtypes Following Maltreatment

The existing literature indicates that sex can "*impact both the nature and severity of psychiatric outcome following maltreatment*", and that "*maltreatment-related psychiatric disorders are associated with a greater prevalence of internalizing psychopathology in females, and greater prevalence of externalizing psychopathology in males*". At the request of one of the reviewers, we have examined this potential effect by running a sex by maltreatment post-hoc analysis to predict Internalizing and Externalizing CBCL sub-scores, respectively. For the internalizing psychopathology, the analysis revealed a main effect of maltreatment ($F(1,824)=151.64$, $p<.001$, $\eta_p^2=.155$; Figure 3A). No significant main effect of sex ($F(1,824)=.004$, $p=.94$, $\eta_p^2=5.311\text{e-}6$) or interaction between sex and maltreatment were found ($F(1,824)=.011$, $p=.73$, $\eta_p^2=1.381\text{e-}4$). For the externalising psychopathology, a similar pattern of results was observed – a main effect of

maltreatment (F(1,824)=120.26, p<.001, η_p^2=.127), a non-significant main effect of sex (F(1,824)=.05, p=.82, η_p^2=6.008e-5), and a non-significant interaction between sex and maltreatment (F(1,824)=.04, p=.83, η_p^2=5.493e-5; Figure 3B).

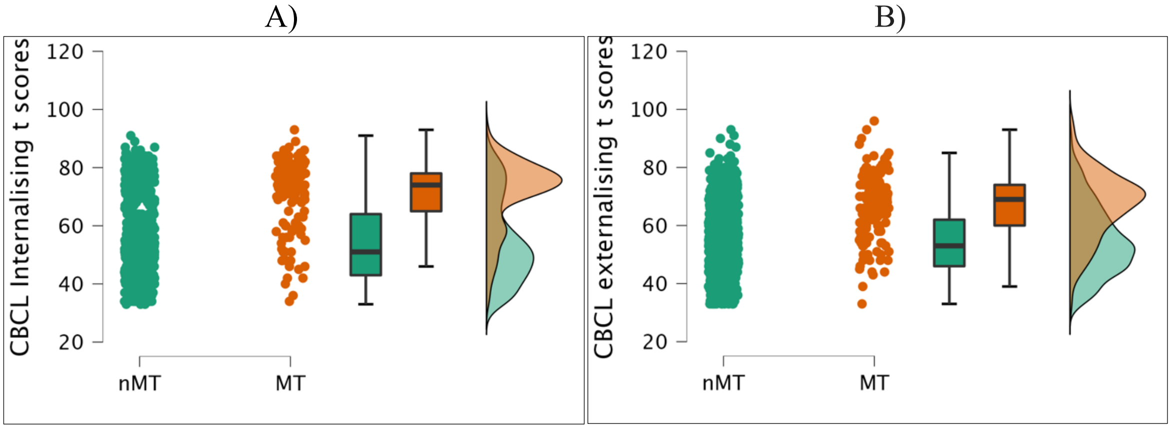

Fig 3. Maltreatment effects on internalising and externalising psychopathology

Supplement 20. ER Analysis by Internalizing and Externalizing Subtypes of Psychopathology

TABLE 6. Main and Interactive Effects of CM and *Internalizing* Psychopathology on Emotion Recognition

**Emotion Recognition**					
Main effects	***F***	***df***	***p***	η_p^2	***Post-hoc comparisons***
**Psychopathology**	15.14	1, 817	*p*<.001	.018	Low > High
**Sex**	16.54	1,817	*p*<.001	.020	Females > Males
Covariates					
**Pubertal category**	33.92	1,817	*p*<.001	.040	-
**SES**	25.31	1,817	*p*<.001	.030	-
**IQ**	15.46	1,817	*p*<.001	.019	-
2-Way Interactions					
**Emotion by Sex**	7.59	5, 4085	*p*<.001	.009	**Disgust**: Females > Males
3-Way Interactions					
**Emotion by Sex by Maltreatment**	2.33	5, 4085	.039	.003	**Disgust:** Maltreatment:

Females > Males

Notes: Covariates evaluated in the model were SES = 0.125, mean-centered IQ=1.245, and pubertal status (1 = pre/early puberty; 2 = mid/late/post puberty). The adjustment for multiple comparisons was obtained using the Bonferroni correction.

TABLE 7. Main and Interactive Effects of CM and *Externalizing* Psychopathology on Emotion Recognition

		Emotion Recognition			
Main effects	***F***	***df***	***p***	η_p^2	***Post-hoc comparisons***
**Maltreatment**	7.84	1, 817	.005	.010	nMT > MT
**Sex**	31.42	1, 817	*p*<.001	.037	Females > Males
Covariates					
**Pubertal category**	34.35	1,817	*p*<.001	.040	-
**SES**	34.29	1,817	*p*<.001	.040	-
**IQ**	22.55	1,817	*p*<.001	.027	-
2-Way Interactions					
**Emotion by Sex**	8.52	5, 4085	*p*<.001	.010	**Sadness, Disgust**: Females > Males
3-Way Interactions					
**Emotion by Sex by Maltreatment**	2.33	5, 4085	.040	.003	**Disgust**: Maltreatment: Females >Males No Maltreatment: Females >Males

Notes: Covariates evaluated in the model were SES = 0.125, mean-centered IQ=1.245, and pubertal status (1 = pre/early puberty; 2 = mid/late/post puberty). The adjustment for multiple comparisons was obtained using the Bonferroni correction.

Supplement 21: Participants' Comorbidities in the Psychopathology Groups

	High Psychopathology	Psychopathology + Maltreatment
Current Diagnoses (in %)	**N= 172**	**N=110**
**ADHD**	46.51	50.9
**ODD**	78.48	90
**DMDD**	2.32	2.72
**SUD**	14.53	14.54
**Anxiety**	13.37	18.18
**OCD**	2.32	3.63
**TIC**	1.74	0.9
**PTSD**	6.39	15.45
**Elimination Disorders**	4.65	7.27
**Eating Disorders**	0.57	0.9
**BPD**	0	0
Lifetime Diagnoses (in %)		
**ADHD**		
**ODD**	3.48	90.9
**DMDD**	2.32	2.72
**SUD**	18.60	19.09
**Anxiety**	20.93	21.81
**OCD**	2.32	3.63
**TIC**	3.48	3.63
**PTSD**	8.13	25.45
**Elimination Disorders**	12.2	25.45
**Eating Disorders**	1.16	1.81
**BPD**	0	0

Notes: ADHD, attention-deficit/hyperactivity disorder; ODD, oppositional defiant disorder; DMDD, disruptive mood dysregulation disorder SUD, substance use disorder; OCD, obsessive–compulsive disorder; PTSD, post-traumatic stress disorder; elimination disorders = enuresis/ encopresis; BPD, borderline personality disorder.

References

Dodge, K. A., Bates, J. E., & Pettit, G. S. (1990). Mechanisms in the cycle of violence. *Science*, 250(4988), 1678-1683.

Eekhout, I., De Vet, H. C., Twisk, J. W., Brand, J. P., de Boer, M. R., & Heymans, M. W. (2014). Missing data in a multi-item instrument were best handled by multiple imputation at the item score level. *Journal of clinical epidemiology*, *67*(3), 335-342.

Kaufman, J., Birmaher, B., Brent, D., Rao, U. M. A., Flynn, C., Moreci, P., ... & Ryan, N. (1997). Schedule for affective disorders and schizophrenia for school-age children-present and lifetime version (K-SADS-PL): initial reliability and validity data. *Journal of the American Academy of Child & Adolescent Psychiatry*, *36*(7), 980-988.

Lansford, J. E., Dodge, K. A., Pettit, G. S., Bates, J. E., Crozier, J., & Kaplow, J. (2002). A 12-year prospective study of the long-term effects of early child physical maltreatment on psychological, behavioral, and academic problems in adolescence. *Archives of Pediatrics and Adolescent Medicine*, *156*(8), 824-830. https://doi.org/10.1001/archpedi.156.8.824

Liu, Y., & De, A. (2015). Multiple imputation by fully conditional specification for dealing with missing data in a large epidemiologic study. *International journal of statistics in medical research*, *4*(3), 287.

Michalos, A. C. (Ed.). (2014). *Encyclopedia of quality of life and well-being research* (pp. 311-1). Dordrecht: Springer Netherlands.

Unesco Institute for Statistics. (2014). ISCED Fields of Education and Training 2013 (ISCED-F 2013): manual to accompany the International Standard Classification of Education.
